# BRD3 and BRD4 BET Bromodomain Proteins Differentially Regulate Skeletal Myogenesis

**DOI:** 10.1038/s41598-017-06483-7

**Published:** 2017-07-21

**Authors:** Thomas C. Roberts, Usue Etxaniz, Alessandra Dall’Agnese, Shwu-Yuan Wu, Cheng-Ming Chiang, Paul E. Brennan, Matthew J. A. Wood, Pier Lorenzo Puri

**Affiliations:** 1 Sanford Burnham Prebys Medical Discovery Institute, Development, Aging and Regeneration Program, La Jolla, CA 92037 USA; 2 Department of Physiology, Anatomy and Genetics, University of Oxford, South Parks Road, Oxford, OX1 3QX UK; 3 Simmons Comprehensive Cancer Center, University of Texas Southwestern Medical Center, 5323 Harry Hines Boulevard, Dallas, Texas 75390 USA; 4 Department of Biochemistry, University of Texas Southwestern Medical Center, 5323 Harry Hines Boulevard, Dallas, Texas 75390 USA; 5 Department of Pharmacology, University of Texas Southwestern Medical Center, 5323 Harry Hines Boulevard, Dallas, Texas 75390 USA; 6 Structural Genomics Consortium and Target Discovery Institute, Nuffield Department of Clinical Medicine, University of Oxford, Oxford, OX3 7DQ UK; 7 IRCCS Fondazione Santa Lucia, Rome, Italy

## Abstract

Myogenic differentiation proceeds through a highly coordinated cascade of gene activation that necessitates epigenomic changes in chromatin structure. Using a screen of small molecule epigenetic probes we identified three compounds which inhibited myogenic differentiation in C2C12 myoblasts; (+)-JQ1, PFI-1, and Bromosporine. These molecules target Bromodomain and Extra Terminal domain (BET) proteins, which are epigenetic readers of acetylated histone lysine tail residues. BETi-mediated anti-myogenic effects were also observed in a model of MYOD1-mediated myogenic conversion of human fibroblasts, and in primary mouse and human myoblasts. All three BET proteins BRD2, BRD3 and BRD4 exhibited distinct and dynamic patterns of protein expression over the course of differentiation without concomitant changes in mRNA levels, suggesting that BET proteins are regulated at the post-transcriptional level. Specific BET protein knockdown by RNA interference revealed that BRD4 was required for myogenic differentiation, whereas BRD3 down-regulation resulted in enhanced myogenic differentiation. ChIP experiments revealed a preferential binding of BRD4 to the *Myog* promoter during C2C12 myoblast differentiation, co-incident with increased levels of H3K27 acetylation. These results have identified an essential role for BET proteins in the regulation of skeletal myogenesis, and assign distinct functions to BRD3 and BRD4.

## Introduction

Skeletal myogenesis is the process whereby mononuclear precursor myoblasts undergo differentiation and fuse to form multinucleated myotubes. This process supports muscle formation throughout development, and during the regeneration of injured or diseased muscle in adult life. Myogenic differentiation requires the sequential activation of genes which constitute the myogenic transcription program, a tightly controlled process involving the interplay between myogenic transcription factors, regulatory non-coding RNAs, and epigenetic changes (including histone modifications, alteration of chromatin compaction, shifts in nucleosome positioning, and DNA methylation)^1–3^.

Post-translational modification of histone tails regulates the accessibility of the genome to the transcriptional apparatus by controlling physical compaction and through recruitment of specific protein cofactors (the so-called histone code hypothesis)^4^. For example, the acetylation of the lysine side-chains on histones H3 and H4 causes chromatin de-compaction and recruitment of proteins containing bromodomain motifs, the end result of which is typically transcriptional activation of the associated genes^5^. Indeed, during skeletal myogenesis, the genome-wide distribution of the myogenic transcriptional activator MYOD1 (MyoD) coincides with peaks of histone hyperacetylation^6^, which are generated by the combined activity of histone acetyltransferases (HATs, including p300/CBP and PCAF) and histone deacetylases (HDACs)^7–9^. This knowledge has inspired pharmacological interventions which promote skeletal myogenesis with epigenetic drugs that target histone acetylation, such as HDAC inhibitors (HDACi)^10, 11^. In particular, acetylation of lysine 27 on histone H3 (H3K27Ac) by HATs is a key modification that promotes enhancer activation during cellular differentiation, including activation of muscle-specific enhancers by MYOD1 in myoblasts^12^. Acetylated histone lysine tail residues are recognized by epigenetic ‘reader’ proteins, such as the Bromodomain and Extra Terminal domain (BET) protein family (consisting of BRD2, BRD3, BRD4, and BRDT) which all contain a pair of bromodomain motifs (BD1 and BD2)^13^. The best studied BET protein, BRD4, remains bound to chromatin during mitosis, promotes cell cycle progression^14, 15^, functions as a transcriptional regulator controlling the release of paused RNA polymerase II via Positive Transcription Elongation Factor b (P-TEFb)^16, 17^, and likely regulates enhancer function through interaction with the Mediator complex^18–21^. BRD4 therefore acts as a physical link between acetylated (i.e. activated) enhancers and the transcriptional machinery.

Inhibition of BRD4 results in downregulation of the *MYC* oncogene and growth arrest^22, 23^, and so BET inhibitors (BETi) are currently under investigation for the treatment of various cancer types in pre-clinical models^22, 24, 25^, and in Phase I/II trials for NUT Midline Carcinoma, Acute Myeloid Leukemia and other hematological malignancies^26^. BET proteins have also been implicated in physiological processes such as inflammation^17, 27, 28^, hematopoiesis^29, 30^, oligodendrocyte differentiation^31^, adipogenesis^32^, spermatogenesis^33^, keratinocyte differentiation^34^, and memory formation^35^. As such, BETi compounds are potential epigenetic modulators of skeletal myogenesis, by targeting events downstream of pro-myogenic enhancer/promoter hyperacetylation. However, the role of BET bromodomain proteins in the regulation of skeletal muscle biology, and the potential effects of BETi on skeletal myogenesis has not been directly addressed to date. Here we have investigated epigenetic regulators of myogenic differentiation using a small molecule inhibitor approach, leading to the discovery of BETi compounds as potent negative modulators of skeletal myogenesis. Further experimentation revealed the individual contributions of BRD3 and BRD4 in the reciprocal regulation of the myoblast-to-myotube transition.

## Results

### Myogenic differentiation is inhibited by BETi compounds

To investigate epigenetic processes contributing to myogenic differentiation we performed a small molecule inhibitor screen using the epigenetic probe library provided by the Structural Genomics Consortium (University of Oxford) (Supplementary Fig. S1a). C2C12 mouse myoblast cells were cultured in Growth Media (GM) for 48 hours and then switched to low serum Differentiation Media (DM) containing epigenetic inhibitor compounds for a further 72 hours. Myogenic differentiation was assessed by Myosin Heavy Chain (MHC) immunostaining and compared with untreated (DM only) and DMSO-treated controls (Supplementary Fig. S1b). Notably, three compounds targeting BET bromodomain proteins inhibited the formation of MHC-positive myotubes. Two of the BET inhibitors are acetyl-lysine mimetics specific to the BET bromodomain family; (+)-JQ1 and PFI-1, whereas the third compound; Bromosporine, is a pan-Bromodomain inhibitor which also binds to CECR2, TAF1, BRD9 and CREBBP, in addition to the proteins of the BET family. The overlap in target specificity suggests that the anti-myogenic effects induced by these structurally dissimilar compounds (triazolothienodiazapine, dihydroquinazoline and triazolopyridazine for (+)-JQ1, PFI-1 and Bromosporine respectively) (Supplementary Fig. S2) are due to the targeting of one or more BET family proteins, and are unlikely to be the result of an off-target effect. Notably, other small molecule inhibitors which exclusively target non-BET bromodomain proteins (i.e. GSK2801, SGC-CBP30, I-CBP112, PFI-3 and C646 targeting BAZ2A/B, CREBBP/EP300, SMARCA2, SMARCA4 and PBRM1) did not induce similar anti-myogenic effects, at least within the range of concentrations used.

**Figure 1:**
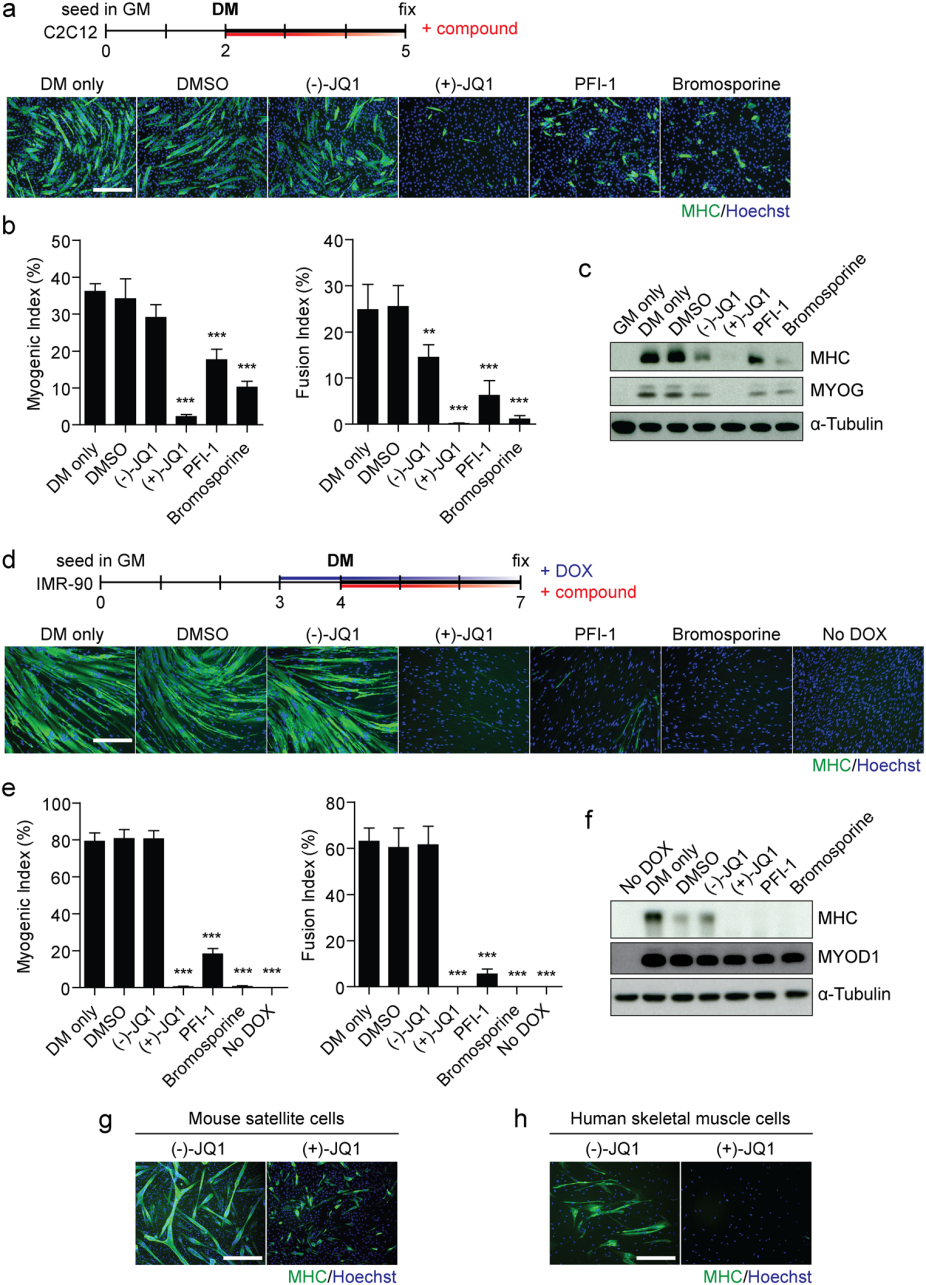
Myogenic differentiation is inhibited by BETi compounds. C2C12 myoblasts were cultured in GM for 2 days and then switched to DM for 3 days. Cultures were treated with BET inhibitors; 10 µM (+)-JQ1, 1 µM PFI-1, and 1 µM Bromosporine at the same time as switching to DM. Untreated (DM only), DMSO-treated, and 10 µM (−)-JQ1-treated cultures were used as negative controls. (**a**) Myogenic differentiation was assessed by immunofluorescence (IF) staining for Myosin Heavy Chain (MHC), and (**b**) quantified using Myogenic and Fusion Indices. (**c**) MHC and MYOG protein expression was determined by Western blot. IMR-90 fibroblasts were treated with doxycycline (DOX) to induce MYOD1 expression and then switched to DM to induce differentiation. Cultures were treated with; 100 nM (+)-JQ1, 1 µM PFI-1, and 1 µM Bromosporine, or controls at the same time as switching to DM. (D) Myogenic differentiation was assessed by (**d**) MHC IF staining and (**e**) Myogenic and Fusion Indices. (**f**) MHC and MYOD1 protein expression was determined by Western blot. (**g**) Primary mouse satellite cells and (**h**) primary human skeletal muscle cells were treated with 1 µM (+)-JQ1 or (−)-JQ1 negative control. Myogenic differentiation was assessed by MHC IF. All microscopy images were taken at 10 × magnification, scale bars indicate 50 µm. Blots were cropped for conciseness and clarity. All values are mean + SD, *n* = 4 representative fields of view, **P < 0.01, ***P < 0.001. Statistical significance was determined by one-way ANOVA with Bonferroni *post hoc* test, and comparisons to the (−)-JQ1 control group reported.

Results with the three BETi compounds were repeated in independent experiments which included (−)-JQ1 as an additional negative control. (−)-JQ1 is the biologically inactive stereoisomer of (+)-JQ1 (Supplementary Fig. S2). All three BETi compounds inhibited myogenic differentiation as assessed by MHC immunofluorescence staining (Fig. 1a) and quantified using Myogenic and Fusion Indices (Fig. 1b). The anti-myogenic effect was strongest for (+)-JQ1 (93% and 99% reduction for Myogenic and Fusion Indices respectively, both *P* < 0.001). Conversely, PFI-1 exerted a weaker effect than the other two compounds (49% and 75% for Myogenic and Fusion Indices respectively, both *P* < 0.001). Cultures treated with highest concentration, 10 µM, (−)-JQ1 did not exhibit the same pronounced anti-myogenic phenotype, although a modest reduction in Fusion Index (43% reduction, *P* < 0.01) and MHC protein expression was observed. The two enantiomers of JQ1 differ only in the orientation of the butyl ester group at the chiral C6 position of the diazepine ring (Supplementary Fig. S2). This alternative orientation is expected to result in a steric clash between (−)-JQ1 and amino acid side chains within the Bromodomain motif. However, given the inherent flexibility of many protein structures; it is not that that surprising that some activity is observed with very high (−)-JQ1 concentrations. Expression of MHC protein was reduced in each of the treatment groups although the effect was strongest for (+)-JQ1 and weakest for PFI-1 (Fig. 1c). Similarly, myogenin (MYOG) protein was undetectable after treatment with (+)-JQ1 (Fig. 1c).

Given that *Myog* is a direct downstream target of the myogenic activator protein MYOD1^36^, we tested whether BET inhibition would be sufficient to prevent the activation of the myogenic program in fibroblasts undergoing MYOD1-mediated conversion to the myogenic lineage. To this end, we utilized IMR-90 human fibroblasts stably transfected with a doxycycline-inducible *Myod1* transgene^37^. Myogenic differentiation was induced in these cells by administration of doxycycline for 24 hours to activate MYOD1 expression, and then switched to DM containing doxycycline and BETi compounds (or control treatments) for a further 72 hours. BETi-treated cultures were almost completely devoid of MHC-positive cells (Fig. 1d) and Myogenic/Fusion Indices were similarly reduced by >99% (*P* < 0.001) for (+)-JQ1 and Bromosporine, and reduced by >77% (*P* < 0.001) for PFI-1 (Fig. 1e). Similarly, MHC protein was undetectable by Western blot after treatment with each BETi compound (Fig. 1f).

As (+)-JQ1 was found to be the BETi compound with the most potent anti-myogenic activity, we selected this compound for further studies in primary cell cultures. Myogenic differentiation was thus also found to be inhibited by (+)-JQ1 treatment in primary satellite cells harvested from the hindlimb muscles of C5BL/6 (wild-type) mice (Fig. 1g), and in primary human skeletal muscle cells (Fig. 1h).

### BETi-mediated inhibition of myogenesis is dose-dependent

**Figure 2:**
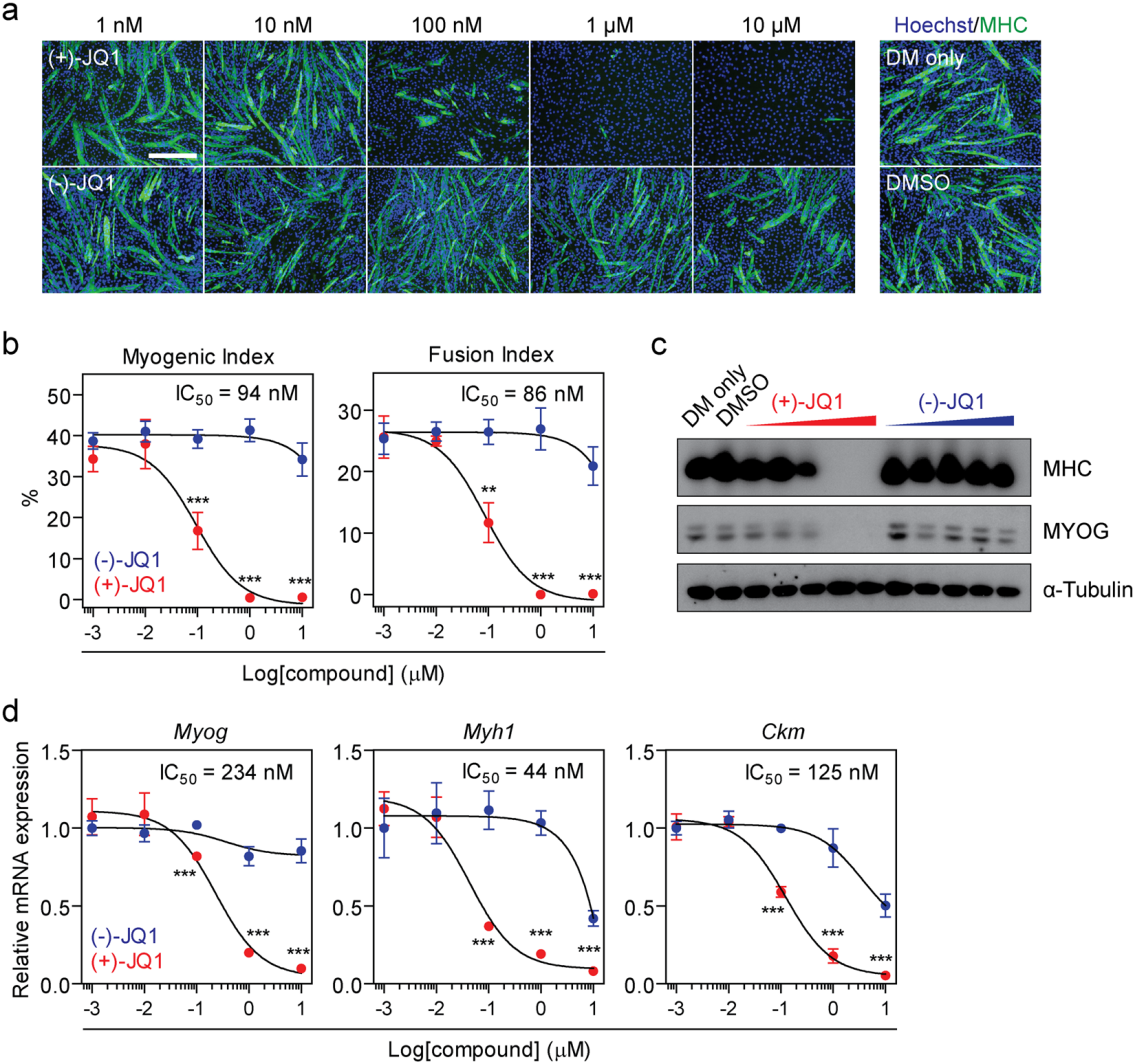
Inhibition of myogenic differentiation by (+)-JQ1 is dose-dependent. C2C12 cultures were treated with (+)-JQ1, or (−)-JQ1 negative control enantiomer, over a range of concentrations (1 nM to 10 µM) and myogenic differentiation assessed by (**a**) MHC IF staining, and (**b**) quantified using Myogenic and Fusion Indices. (**c**) MHC and MYOG protein levels were determined by Western blot. (**d**) *Myh1*, *Myog* and *Ckm* mRNA levels were determined by RT-qPCR. All images were taken at 10× magnification, scale bars indicate 50 µm. Blots were cropped for conciseness and clarity. All values are mean +/− SD, *n* = 4 representative fields of view for microscopy, *n* = 3 for RT-qPCR, **P < 0.01, ***P < 0.001 (unpaired *t*-test comparing (+)-JQ1 and (−)-JQ1 at each time point).

To test the dose-dependence of C2C12 differentiation to (+)-JQ1 treatment, a range of compound concentrations (1 nM to 10 µM) were tested and anti-myogenic activity compared to (−)-JQ1 controls. Myogenic inhibition was found to be highly dose-dependent as determined by MHC immunofluorescence (Fig. 2a) and quantified by Myogenic/Fusion Indices (Fig. 2b). Similarly, dose-dependent reductions in MHC and MYOG protein levels (Fig. 2c), and *Myh1*, *Myog*, and *Ckm* transcript levels (Fig. 2d), were observed by Western blot and RT-qPCR respectively. In all cases, (+)-JQ1 activity reached a plateau of maximal effect at 1 µM and was ineffective at 10 nM. IC_50_ values for (+)-JQ1 ranged from 44 nM to 234 nM (Fig. 2b,c,d). At 1 µM, both Myogenic and Fusion Indices were reduced by >99% (*P* < 0.001), and expression of all three myogenic transcripts were reduced by >76% (*P* < 0.001). Importantly, at this concentration no activity was observed with the negative control compound (−)-JQ1 (Fig. 2b,c,d).

### BETi treatment promotes G1 arrest in differentiating myoblasts

**Figure 3:**
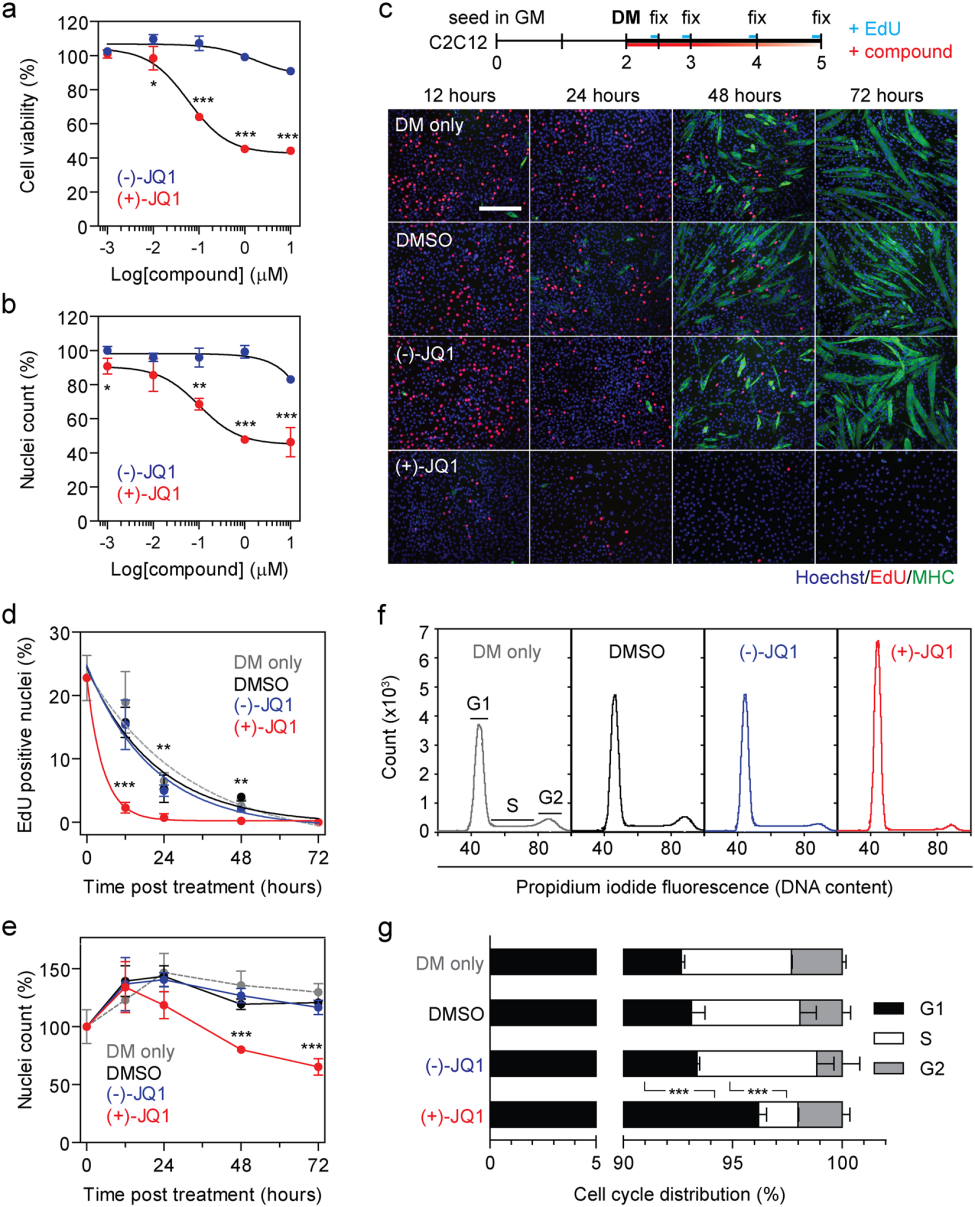
(+)-JQ1 inhibits the cell cycle by G1 arrest in differentiating myoblasts. C2C12 cultures were treated with (+)-JQ1, or the (−)-JQ1 inactive enantiomer, over a range of concentrations (1 nM to 10 µM) and cell viability assessed by (**a**) MTS assay, and (**b**) nuclei counting. Cell cycle progression was assessed by EdU incorporation assay. (**c**) C2C12 cultures were switched to DM and treated with 1 µM (+)-JQ1, (−)-JQ1, DMSO or untreated (DM only). Cells were fixed at 12, 24, 48 and 72 hours after treatment and pulsed with EdU 2 hours before fixation. EdU positive nuclei were stained with Alexa Fluor 555 and cultures immunostained for MHC. (**d**) The percentage of EdU positive nuclei was determined by cell counting. (**e**) Changes in the number of nuclei over time, and in response to compound treatment were determined by counting nuclei. (**f**) The effect of 1 µM (+)-JQ1 on cell cycle progression was determined by flow cytometry analysis of DNA content, (**g**) and the proportions of nuclei in G1, S and G2 phases quantified. Images were taken at 10 × magnification, scale bars indicate 50 µm. Values are mean +/− SD, *n* = 4 representative fields of view for microscopy, *n* = 6 for MTS data, *n* = 3 cultures for flow cytometry (30,000 singlet events counted per sample), *P < 0.05, **P < 0.01, ***P < 0.001 (Comparisons between two groups were tested using an unpaired *t*-test. Comparisons between multiple groups were tested using one-way ANOVA with Bonferroni *post hoc* test, and the result for the (−)-JQ1 versus (+)-JQ1 comparison reported).

A dose-dependent reduction in cell viability (Fig. 3a) and the number of nuclei per field of view (Fig. 3b) was observed in C2C12 myoblasts treated with (+)-JQ1 (but not for the (−)-JQ1 control) that closely mirrored the anti-myogenic phenotype. We reasoned that this effect could be the result of either a reduction in proliferation or an increase in cell death. To investigate this further, cell proliferation was monitored using an EdU (5-ethynyl-2′-deoxyuridine) incorporation assay. C2C12 cells treated with DMSO, (−)-JQ1, (+)-JQ1, or left untreated (DM only) were cultured for 12, 24, 48, and 72 hours after treatment in DM. Cultures were pulsed with 10 µM EdU for 2 hours before fixation at each time point. The number of cells that incorporated EdU gradually declined over time after switching to DM, as myoblasts in the control groups withdrew from the cell cycle and differentiated to form MHC-positive myotubes (Fig. 3c,d). In contrast, the (+)-JQ1-treated group showed a marked reduction (85%, *P* < 0.001) in EdU-positive cells after 12 hours in DM, indicative of a strong anti-proliferative effect in the absence of myotube formation (Fig. 3c,d). The number of nuclei per field of view increased above the baseline for all groups before reaching a plateau at 12 hours after switching to DM. Treatment with (+)-JQ1 resulted in a reduction in nuclei numbers relative to controls at all subsequent time points (>37% reduction, *P* < 0.001) (Fig. 3e). Assessment of cell cycle status by flow cytometry 12 hours after treatment showed that (+)-JQ1 induced arrest in G1 (Fig. 3f,g), consistent with previous reports^14, 15, 22, 38^. Conversely, to assess cell death, treated cultures were stained with Annexin V-FITC and propidium iodide (to label early-stage apoptotic cells and late-stage apoptotic/necrotic cells respectively). C2C12 cells treated with (+)-JQ1 exhibited minimal staining relative to negative controls (Supplementary Fig. S3). Together, these data suggest that treatment of C2C12 cells with (+)-JQ1 inhibits myoblast proliferation by G1 arrest, with minimal induction of apoptosis/necrosis.

### Effect of BET inhibition prior to exposure to differentiation stimulus

**Figure 4:**
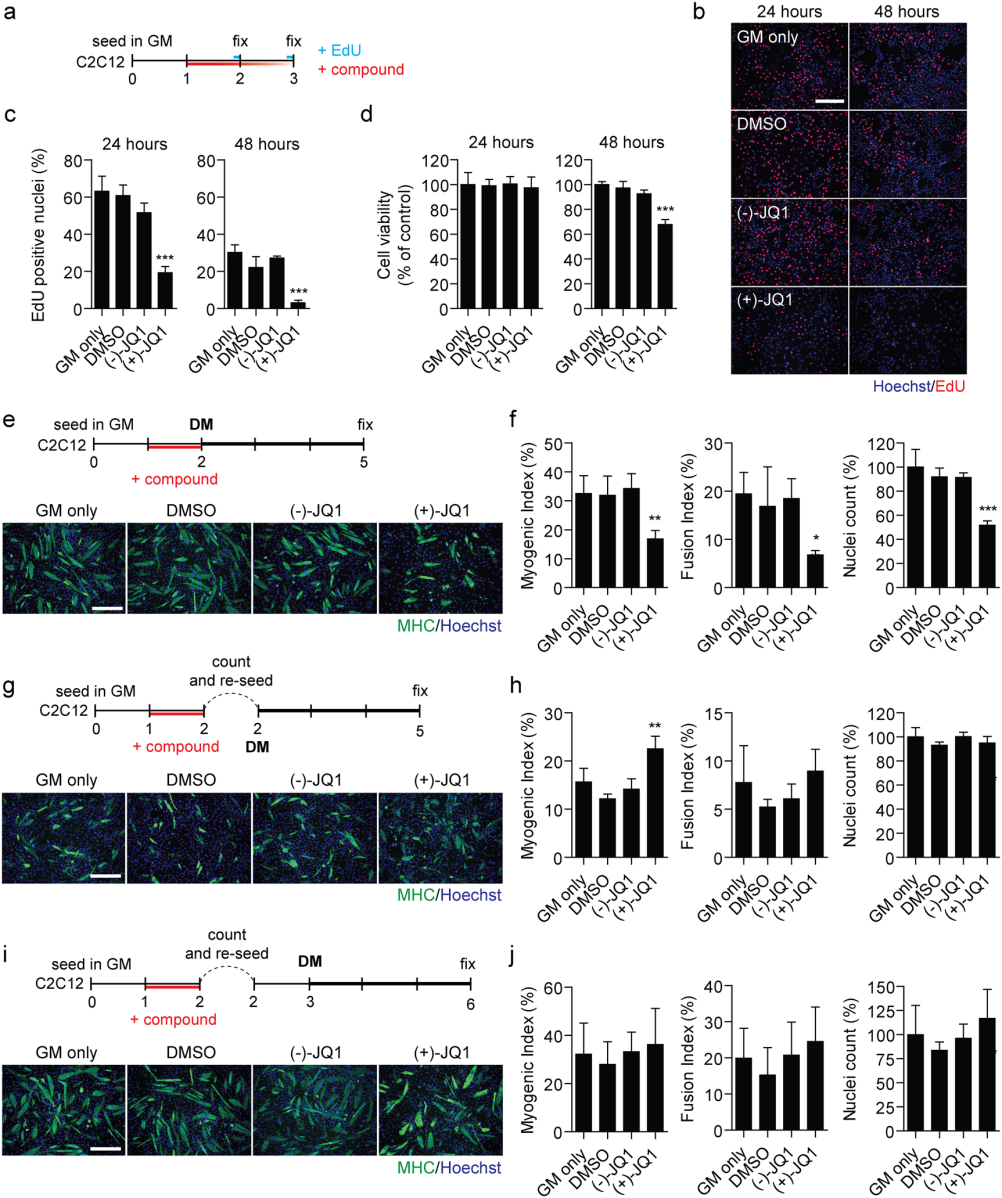
Effect of BET inhibition in growth media. (**a**) C2C12 myoblasts were cultured in GM and treated after 24 hours with 1 µM (+)-JQ1 or controls; (−)-JQ1 inactive stereoisomer, DMSO, or untreated (GM only). Cells were harvested 24 and 48 hours after treatment. Prior to fixing cells were pulsed with EdU for 2 hours to label proliferating cells. (**b**) EdU incorporation was visualized by fluorescence microscopy, and (**c**) the percentage of EdU positive cells determined by cell counting. (**d**) In parallel, cell viability was assessed by MTS assay. (**e**) To test the effect of compound treatments prior to the induction of differentiation, C2C12 cells were treated with compounds (as above) in GM for 24 hours and then switched to DM for 3 days. Myogenic differentiation was assessed by MHC IF staining for Myosin Heavy Chain (MHC), and (**f**) Myogenic and Fusion Indices quantified, and nuclei counted. Similar experiments were further performed to control for cell density. (**g,h**) Firstly, C2C12 cells were treated for 24 hours in GM, collected by trypsinization and cells counted. Equal numbers of cells (2 × 10^5^ cells per well of a 24 well plate) from each treatment group were re-seeded in DM and cultured for a further three days before fixing. (**i**,**j**) Secondly, C2C12 cells were treated in GM for 24 hours, trypsinized, and counted as described above. Equal numbers of cells were re-seeded in GM for an additional 24 hours, before switching to DM for a further 3 days. Images were taken at 10× magnification, scale bars indicate 50 µm. Values are mean +/− SD, *n* = 4 representative fields of view for microscopy, *n* = 9 for MTS data, *P < 0.05, **P < 0.01, ***P < 0.001. Statistical significance was determined by one-way ANOVA with Bonferroni *post hoc* test, and the result for the (−)-JQ1 versus (+)-JQ1 comparison reported.

Given the pronounced cell cycle phenotype in differentiating myoblasts, we next sought to investigate the effect of BET inhibition on myoblasts in growth conditions only (i.e. in GM). C2C12 cells were treated with 1 µM (+)-JQ1 (or controls) and cultures harvested after an EdU pulse at 24 and 48 hours post treatment (Fig. 4a). EdU incorporation was reduced by 63% and 89% relative to the negative control enantiomer at 24 and 48 hours post (+)-JQ1 treatment respectively (Fig. 4b,c), indicative of a pronounced anti-proliferative effect similar to that observed in Fig. 3. Notably, cell viability was not affected by exposure to (+)-JQ1 for 24 hours, indicating that the compound is not cytotoxic at this concentration (Fig. 4d). Conversely, cell viability was significantly reduced at 48 hours post treatment (Fig. 4d), likely as a consequence of reduced cellular proliferation in the preceding 48 hours.

To investigate whether BETi treatment in GM could have an impact on the efficiency of myogenic differentiation, C2C12 cultures were treated with (+)-JQ1 (or controls) in GM for 24 hours and then switched to DM for 3 days in the absence of compound (Fig. 4e). (+)-JQ1 treatment in GM resulted in partial inhibition of myogenic differentiation (Fig. 4e,f). Myogenic and Fusion Indices were reduced by 50% and 63% respectively (Fig. 4f). Notably, the magnitude of this inhibition was much less than was observed for BET inhibition in DM (Figs 1, 2 and 3). Importantly, the strong anti-proliferative effect of (+)-JQ1 in GM led to reduced confluence at the time of switching to DM, relative to the control groups; (−)-JQ1, DMSO, and untreated (GM only) (Fig. 4f).

We reasoned that this reduced cell density might account for anti-myogenic effect observed with this experimental design. We therefore controlled for cell density, by treating C2C12 cells in GM for 24 hours with (+)-JQ1, trypsinizing, counting, and then re-seeding equal numbers of cells for each experimental group in DM to induce differentiation (Fig. 4g). With this protocol, (+)-JQ1 failed to induce any anti-myogenic effect as typically observed when treatment was initiated in DM. Instead a 48% increase in Myogenic Index was observed (although the Fusion Index was not significantly affected) (Fig. 4g). This positive effect of BETi on myogenic differentiation may be due to the cultures being synchronized in G1 at the time of exposure to DM. To investigate the reversibility of BETi treatment in GM, C2C12 cells were treated as above, and then equal cell numbers re-seeded in GM for 1 further day before switching to DM (Fig. 4i). With this experimental design no significant differences were observed between any of the experimental groups (Fig. 4j), suggesting that the effects of (+)-JQ1 treatment in GM is transient and readily reversed by exposure to pro-mitogenic media (GM), as opposed to differentiation-inducing conditions (DM). Overall, these data show that the effects of (+)-JQ1 observed in GM are due to interference with the cell cycle and reduced cell density.

### BETi treatment in DM inhibits differentiation independently of cell cycle arrest

**Figure 5:**
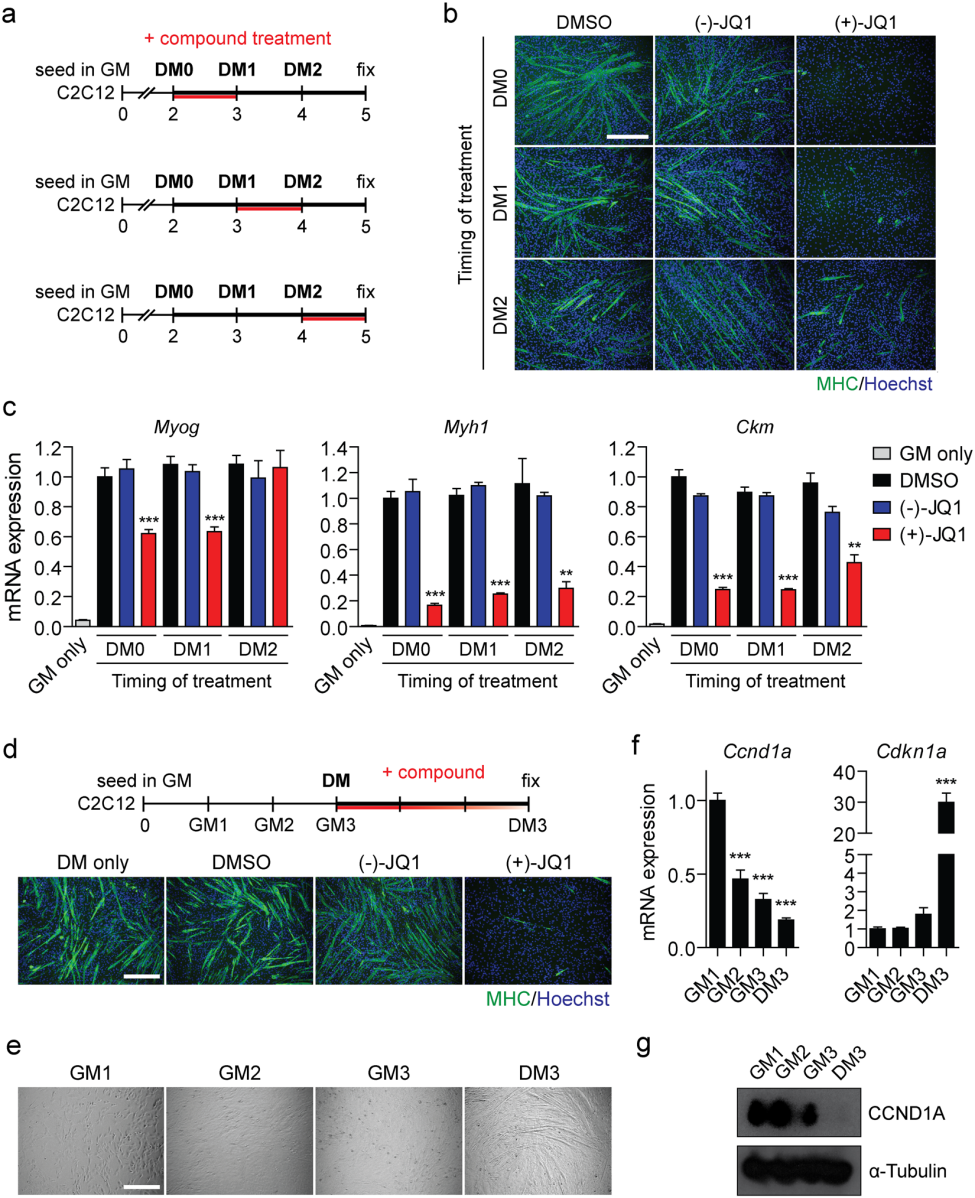
BETi treatment inhibits differentiation independently of cell cycle arrest. (**a**) C2C12 cells were treated with 1 µM (+)-JQ1 for a 24 hour interval starting at day 2, 3 or 4 (corresponding to zero, one or two days in Differentiation Media; DM0, DM1 and DM2). Fresh DM was added at each time point in order to wash out the compound treatments. (**b**) Myogenic differentiation was assessed by MHC IF staining and compared with DMSO and (−)-JQ1-treated negative controls. (**c**) Myogenic transcript levels for *Myog*, *Myh1* and *Ckm* were determined for each treatment protocol by RT-qPCR. Cells treated with GM only were included as an additional undifferentiated control. (**d**) C2C12 cells were cultured for 3 days in GM in order to reach full confluence and promote maximal cell cycle withdrawal prior to switching to DM for 3 days. Cultures were treated with 1 µM (+)-JQ1 concurrent with the switch to DM. Cultures treated with (−)-JQ1, DMSO, or left untreated (DM only) served as negative controls. Cells were fixed, and myogenic differentiation was assessed by MHC IF staining. (**b**) Light microscopy images indicate that cells were fully confluent by day 2 in Growth Media (GM2). (**c**) Changes in the cell cycle markers *Ccnd1a* (Cyclin D1) and *Cdkn1a* (p21) were assessed by RT-qPCR, and (**d**) CCND1A by western blot. All microscopy images were taken at 10× magnification, scale bars indicate 50 µm. Blots were cropped for conciseness and clarity. All values are mean + SD, *n* = 3, **P < 0.01, ***P < 0.001. The mean of the DMSO group (treated at DM0) was scaled to a value of one. Comparisons between multiple groups were tested using one-way ANOVA with Bonferroni *post hoc* test, and comparisons to the (−)-JQ1 or GM1 group reported.

Since irreversible cell cycle withdrawal typically precedes the activation of muscle gene expression during skeletal myogenesis^39^, the G1 arrest during BETi-mediated inhibition of myogenic differentiation in DM appears paradoxical. Indeed, (+)-JQ1-mediated cell cycle arrest can promote differentiation in other lineages (e.g. squamous cell differentiation)^22^. To determine if the anti-proliferative and anti-differentiation effects of (+)-JQ1 are distinct, C2C12 cultures were treated with (+)-JQ1 in a staggered manner whereby cells were exposed to the compound (or controls) for a 24-hour period at the time of the initial switch to DM (DM0), or after the initial differentiation stimulus starting at either day 3 (DM1) or day 4 (DM2) (Fig. 5a). A single 24-hour window of (+)-JQ1 treatment concurrent with the switch to DM was found to be sufficient to inhibit myogenic differentiation to a level equivalent to that observed in previous experiments with 3-day exposures (Fig. 5b). Importantly, treatment with (+)-JQ1 at the later time points after the switch to DM (at a time when the majority of myoblasts have withdrawn from the cell cycle, Fig. 3) also inhibited the formation of MHC-positive myotubes (Fig. 5b). Similarly, all three (+)-JQ1 treatment protocols resulted in reduced expression of *Myh1* and *Ckm* transcripts, although the effect was less pronounced for the later treatment protocol (Fig. 5c). Conversely, *Myog* was reduced by ~40% in the case of the early treatment protocols (DM0 and DM1) but was unchanged in the case of the late treatment protocol (DM2). We interpreted this result as an effect of BETi treatment on late-stage myogenesis that is restricted to genes downstream of MYOG, which was activated during the period of BETi-free DM culture. To further explore the relationship between the anti-myogenic effects of BET inhibition and their influence on the cell cycle, C2C12 cells were cultured for three days in GM to reach high cell confluency before BETi treatment in DM for a further three days. The purpose of this experimental design was to further promote maximal cell cycle withdrawal before the initiation of BET inhibition (Fig. 5d). Using this protocol, (+)-JQ1 treatment still resulted in a profound abrogation of myogenic differentiation (Fig. 5d). Visualization of cultures by light microscopy revealed that cells were completely confluent after two days in GM (Fig. 5e). Furthermore, the cell cycle markers *Ccnd1a* (Cyclin D1) and *Cdkn1a* (p21) were progressively down- and up-regulated respectively as cells became more confluent, reflecting the degree of cell cycle withdrawal at the time of (+)-JQ1 treatment (Fig. 5f,g). Taken together, these experiments indicate that the anti-myogenic phenotype induced by (+)-JQ1 is independent of its anti-proliferative activity.

We next investigated the relationship between the timing of BET inhibition and the relative impact on the activation of muscle gene expression. We again utilized the IMR-90 fibroblast-myoblast conversion model, as the timing of MYOD1 expression can be precisely controlled by the administration of doxycycline. Sequential treatment of IMR-90 cultures with doxycycline, followed by (+)-JQ1 24 hours later resulted in total absence of MHC protein expression (a late-stage marker of myogenic differentiation) whereas MYOG expression, an early-stage marker of myogenic differentiation, was only moderately reduced (Supplementary Fig. 4). Conversely, simultaneous treatment with doxycycline and (+)-JQ1 induced a much stronger reduction in MYOG protein in addition to the total absence of MHC expression (Supplementary Fig. 4). These data show that (+)-JQ1 can inhibit the expression of differentiation-associated genes at distinct stages downstream of induction by MYOD1, as also supported by results from Fig. 5c.

### BETi treatment induces prolonged inhibition of myogenic differentiation

**Figure 6:**
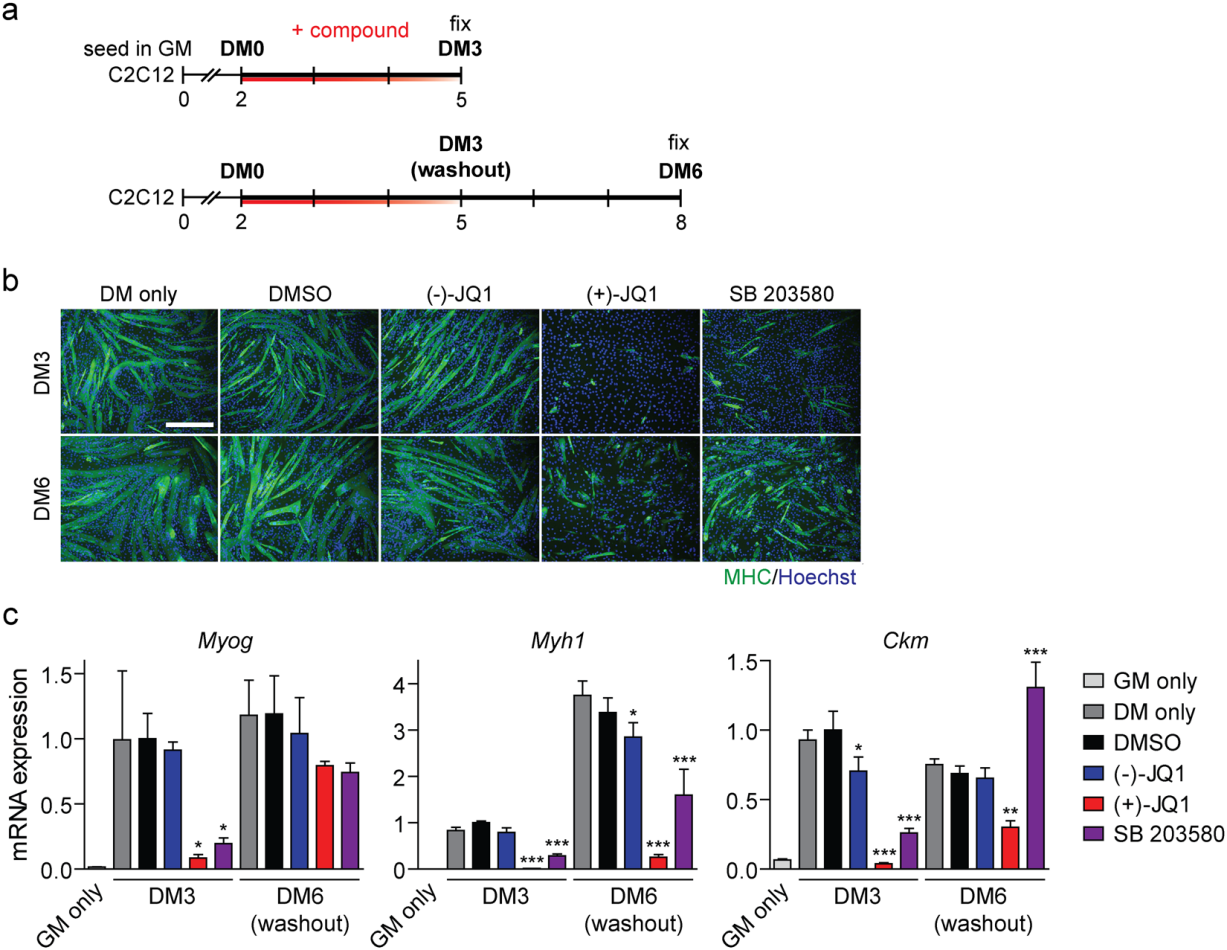
BETi treatment induces prolonged inhibition of myogenic differentiation in differentiation conditions. (**a**) C2C12 cells were treated with 1 µM (+)-JQ1 or 10 µM SB 203580 for a 3 day period in DM and (**b**) cultures either fixed for MHC IF staining or cultured for an additional 3 days in fresh DM to washout the compounds before fixing. Myogenic differentiation was assessed relative to untreated (DM only), DMSO, and (−)-JQ1 negative controls. (**c**) Myogenic transcript levels for *Myog*, *Myh1* and *Ckm* were determined for each treatment protocol by RT-qPCR. Cells treated with GM only were included as an additional undifferentiated control. Images were taken at 10× magnification, scale bars indicate 50 µm. All values are mean + SD, *n* = 3, *P < 0.05, **P < 0.01, ***P < 0.001. The mean of the DMSO group at day 5 was scaled to a value of one. Comparisons between multiple groups were tested using one-way ANOVA with Bonferroni *post hoc* test, and comparisons to the DMSO group reported.

To investigate the duration of the anti-myogenic effect, C2C12 cultures were treated with (+)-JQ1 in DM for three days and then harvested immediately (DM3), or after a further 3 days in culture (DM6) with washout of the compound (Fig. 6a). The effect of (+)-JQ1 was found to be stable up to 6 days after initiation of treatment, with few MHC-positive cells (and only a few sporadic multinucleated myotubes) present. In contrast, cells treated with SB 203580, an inhibitor of p38α/β (MAPK12/MAPK11) that prevents the proliferation-to-differentiation transition^40, 41^, induced a less pronounced inhibition of myogenic differentiation at day DM3, and myogenic differentiation was mostly recovered by day DM6 (Fig. 6b). *Myog*, *Myh1* and *Ckm* transcript levels were determined by RT-qPCR at each time point (Fig. 6c). At DM3, all three transcripts were significantly repressed by treatment with (+)-JQ1 and SB 203580, although the effect was more pronounced for (+)-JQ1. At DM6 (following compound washout), *Myh1* and *Ckm* were still expressed at low levels in the (+)-JQ1 treated group, whereas for the SB 203580-treated cultures *Myh1* was partially recovered and *Ckm* expressed at a level above that of the controls, consistent with the recovery apparent from the MHC immunostaining (Fig. 6b). The different mechanisms of action for these two pharmacological approaches likely account for these data. While p38-mediated phosphorylation is readily reversed, BET genes function via multiple mechanisms, the disruption of which that may require a longer time for reversion. However, we cannot exclude the possibility that (+)-JQ1 remains bound to BET proteins for extended periods of time.

### Time course of BET bromodomain expression in differentiating C2C12 myoblasts

**Figure 7:**
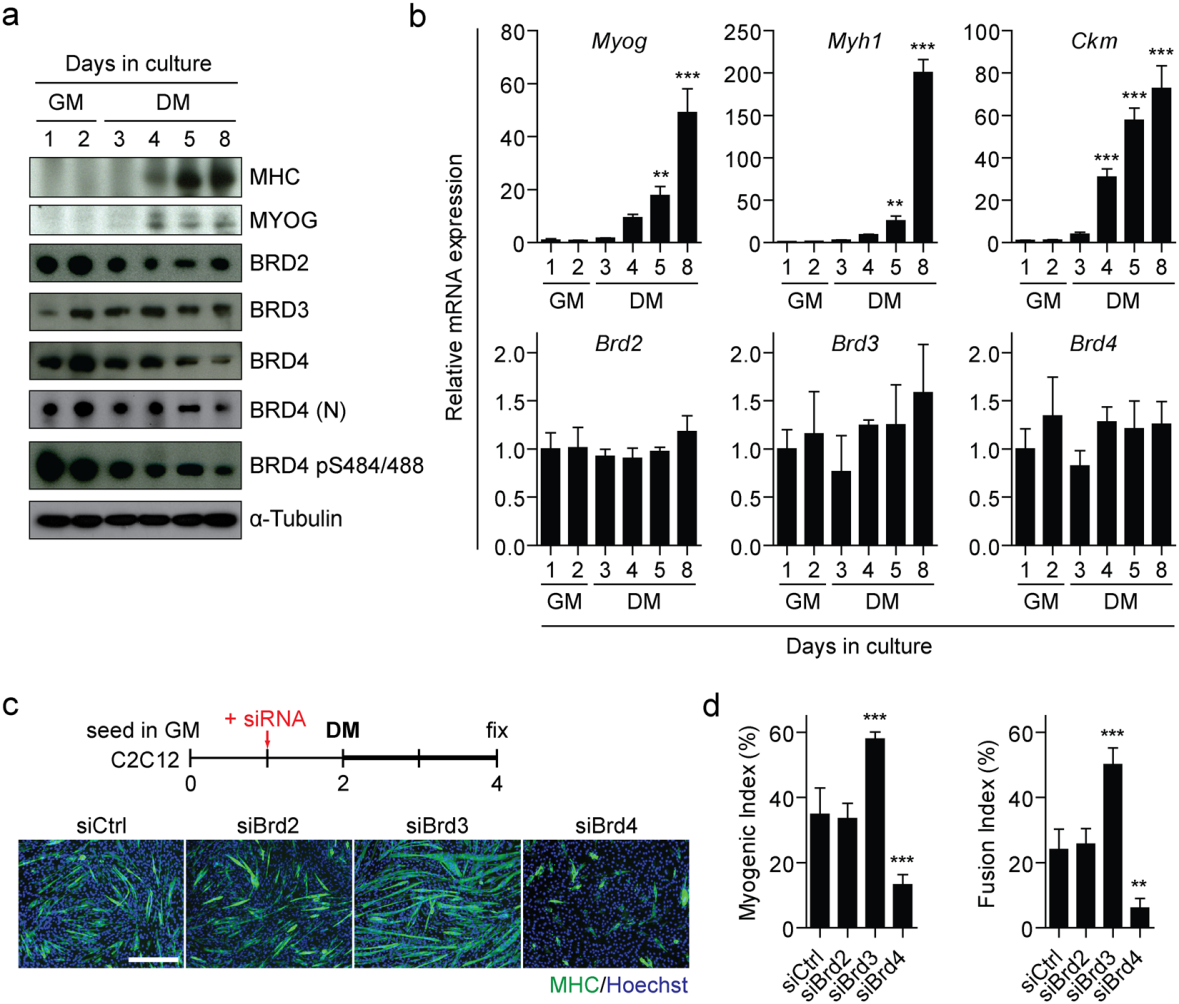
Differential expression and function of BET bromodomain proteins during myogenic differentiation. C2C12 cells were cultured in GM for 2 days followed by DM for a further 6 days and cells harvested at the indicated time points. Expression of myogenic factors and BET bromodomain genes was assessed by (**a**) Western blot, and (**b**) RT-qPCR. Three antibodies were used against BRD4. Total BRD4 levels were determined using two antibodies; anti-BRD4, which is commercially available, and anti-BRD4 (N) which is an in-house antibody directed against the N-terminus. Phosphorylated BRD4 was measured using anti-BRD4 pS484/488. (**c**) C2C12 cultures were treated with 100 nM siBrd3, siBrd4 or the negative control siRNA (siCtrl) in GM. After 24 hours, cultures were switched to DM and cultured for a further 48 hours. Myogenic differentiation was assessed by MHC IF staining and (**d**) quantified using Myogenic and Fusion Indices. Images were taken at 10× magnification, scale bars indicate 50 µm. Blots were cropped for conciseness and clarity. All values are mean + SD, *n* = 4 representative fields of view for microscopy, *n* = 3 for RT-qPCR, **P < 0.01, ***P < 0.001. Statistical significance was determined by one-way ANOVA with Bonferroni *post hoc* test, and comparisons to the GM1 or siCtrl groups reported.

To investigate the role played by individual BET bromodomain proteins in myogenic differentiation, we determined the expression profile of BET proteins in C2C12 cells at various time points during the transition from myoblasts to myotubes. This process is typically accompanied by the induction of muscle-specific proteins; MHC and MYOG, and transcripts; *Myog*, *Myh1* and *Ckm*, upon switching C2C12 cultures to DM (Fig. 7a,b). During this time course, all three BET proteins were dynamically expressed with each individual BET protein exhibiting a distinct expression pattern. BRD2 was expressed at high levels in GM and then stably expressed at lower levels in DM. BRD3 was expressed at very low levels after 1 day in GM, increased after 2 days in GM, and then maintained this level of protein expression in DM. For BRD4, expression increased to high levels at 2 days in GM and then gradually declined as differentiation progressed (similar results were observed with two anti-BRD4 antibodies) (Fig. 7a). Additionally, the expression level of BRD4 phosphorylated on serine residues 484 and 488 (BRD4 pS484/488) was also measured as post-translational modification has been implicated in the regulation of BRD4 function^21, 34, 35, 42^. Phosphorylated BRD4 followed the same general pattern of expression as total BRD4. BRDT (testis/ovaries-specific) is not expressed in muscle and so was not investigated.

In contrast to protein levels, the mRNA levels for *Brd2*, *Brd3*, and *Brd4* were stably expressed throughout the time course (Fig. 7b), suggesting that the dynamic patterns of BET protein expression during myogenic differentiation are likely a consequence of post-transcriptional gene regulation. The three BET mRNA transcripts were expressed at similar levels (average Cq values: 22.7, 23.4, and 22.6 for *Brd2*, *Brd3*, and *Brd4* respectively).

### BRD3 and BRD4 exert opposing effects on myogenic differentiation

To identify which BET bromodomain protein is responsible for the observed reduction in myogenic activity observed with (+)-JQ1 treatment, we depleted BRD3 and BRD4 by RNA interference in C2C12 myoblasts in GM and then induced myogenic differentiation by switching to DM. In the case of BRD2, only minimal protein level knockdown could be achieved (Supplementary Fig. S5), and so this protein was not investigated further. The anti-myogenic phenotype observed following BETi treatment was recapitulated in BRD4 knockdown cultures (62% and 75% reductions in Myogenic and Fusion Indices respectively, *P* < 0.01) suggesting that BRD4 is required for myogenic differentiation (Fig. 7c,d). This profound phenotypic change was observed despite only partial knockdown of BRD4 (Supplementary Fig. S5). Surprisingly, knockdown of BRD3 elicited the opposite phenotype, whereby myogenic differentiation was enhanced (66% and 108% increases for Myogenic and Fusion Indices respectively, *P* < 0.001) (Fig. 7c,d). These findings indicate that BRD3 and BRD4 exert reciprocal regulatory effects on myogenic differentiation.

To investigate a potential direct role for the BET proteins in the regulation of the myogenic transcription program we performed Chromatin Immunoprecipitation (ChIP) with antibodies against H3K27Ac, BRD3 and BRD4. Chromatin was harvested from C2C12 cells cultured for two days in GM, and after a further 3 days in DM. Parallel cultures were treated with (−)-JQ1 or (+)-JQ1 in DM. ChIP enrichment was assessed by qPCR using primers spanning the proximal E-box in the *Myog* promoter, a site which shows increased MYOD1 occupancy as cells differentiate^36, 43^. The transcription start site (TSS) of the *Sox2* gene was used as a putative negative control region as this gene is not expressed in C2C12 cells.

**Figure 8:**
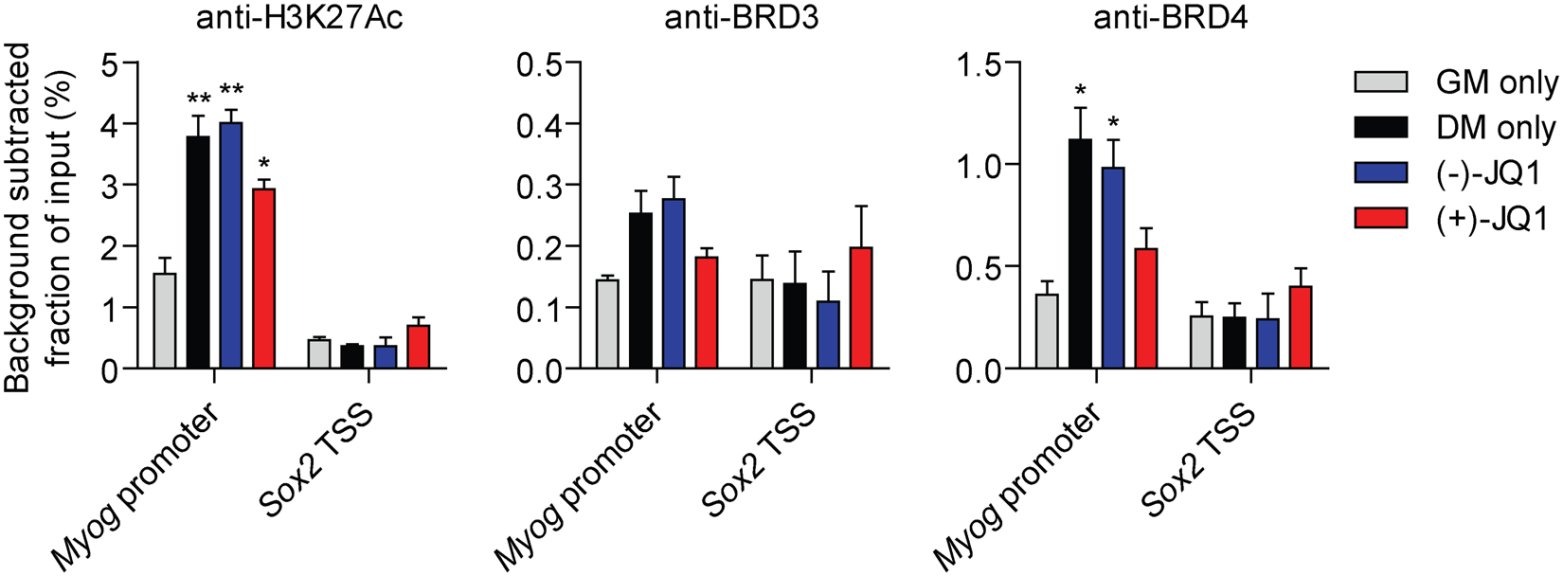
BRD4 is enriched at the *Myog* promoter during myogenic differentiation. Chromatin was harvested from undifferentiated C2C12 cells (two days in GM), differentiated cells (2 days in GM, followed by 3 days in DM), and differentiated cells treated with either (−)-JQ1 or (+)-JQ1 (1 μM). Sonicated chromatin was precipitated with anti-H3K27Ac, anti-BRD3, anti-BRD4 or IgG (negative background control) antibodies. Genomic DNA was amplified by qPCR using primers for the *Myog* promoter or the *Sox2* TSS (a putative negative control locus). Enrichment is expressed as the fraction of non-precipitated input samples after subtraction of the background IgG signal. For BRD3, one way ANOVA analysis was significant (*P* = 0.0399), but no pairwise Bonferroni *post test* reached significance at the *P* < 0.05 level. All values are mean + SEM, *n* = 3 independent experiments, statistical comparisons are to the GM only group, *P < 0.05, **P < 0.01, ***P < 0.001 (one-way ANOVA, Bonferroni *post hoc* test).

Enrichment of H3K27Ac increased by 2.5-fold (*P* < 0.01) at the *Myog* promoter in DM relative to GM consistent with the activation of *Myog* expression in differentiating cells. H3K27Ac was similarly increased by 2.6 fold (*P* < 0.01) after treatment with (−)-JQ1 (Fig. 8). Treatment with (+)-JQ1 resulted in a modest reduction (27%) in H3K27Ac enrichment relative to the (−)-JQ1 control. The enrichment of BRD4 at the *Myog* promoter followed a similar pattern. BRD4 occupancy increased by 3.1-fold (*P* < 0.05) in DM and 2.7-fold (*P* < 0.05) in (−)-JQ1-treated cells relative to GM (Fig. 8). Treatment with (+)-JQ1 decreased BRD4 occupancy (by 40%) relative to the (−)-JQ1 control. For both antibodies low levels of enrichment were observed at the *Sox2* TSS, and there was no significant change in relative enrichment between experimental groups at this locus. BRD3 binding was also detected at the *Myog* promoter, although at much reduced levels which were close to the background occupancy detected at the *Sox2* TSS. The pattern of occupancy was similar to that observed for BRD4 and H3K27Ac, although the percentage enrichment was ~5 fold lower. Together, these findings suggest that BRD4 is recruited to pro-myogenic loci that become hyper-acetylated during differentiation.

## Discussion

Here we have shown that myogenic differentiation is inhibited by BETi compounds in multiple cell models. The most potent molecule tested, (+)-JQ1, exhibited highly dose-dependent anti-myogenic activity with IC_50_ values ~100 nM. (+)-JQ1 treatment also promoted withdrawal from the cell cycle, although this reduction in proliferation was shown to be independent of its anti-differentiation activity. The anti-myogenic effect of pan-BET inhibitors was phenocopied when BRD4 was selectively depleted by RNAi. These data provide the first evidence that BET proteins, and BRD4 in particular, play important roles in myogenic differentiation. Notably, one previous study by Proserpio *et al*. showed that BRD4 interacts with the methyltransferase SMYD3 in order to regulate the transcription of *Mstn* (Myostatin) in the context of muscle atrophy^44^. In this instance, treatment with (+)-JQ1 was shown to promote myogenic differentiation and repress the expression of atrophy-associated transcripts^44^. Importantly, in the experiment performed by Proserpio *et al*.^44^, treatment with (+)-JQ1 was initiated in GM, and was used at a concentration below that found to be effective in the studies presented here.

BRD4 is known to be involved in transcriptional control by regulating the release of paused RNA polymerase II via interaction with P-TEFb, and by association with the Mediator complex. As such, BRD4 has previously been shown to act as a coactivator of specific subsets of genes in the context of NF-κB-mediated inflammation^27, 28^. Here we have demonstrated that BRD4 binds to the *Myog* locus, and that binding is impaired by treatment with (+)-JQ1. It is therefore possible that BRD4 is involved in the activation of the muscle transcription program by binding to hyperacetylated pro-myogenic enhancers and/or promoters. Interestingly, treatment with (+)-JQ1 also induced a modest decrease in *Myog* promoter acetylation, suggesting that BRD4 may play a role in maintaining a transcriptionally active chromatin structure, perhaps via interactions with histone acetyltransferases.

Several studies have identified distinct roles for the two bromodomain motifs found in all BET family members^29–31^. A common theme is that the bromodomains may differentially interact with acetylated histones, acetylated non-histone proteins, or may act to cooperatively bind to both. Notably, BRD3 has been shown to bind the erythroid transcription factor GATA1 via BD1^29, 30^. Given that MYOD1 is activated by CBP/p300 or PCAF mediated-acetylation^45, 46^, we speculate that BRD4 may analogously interact with acetylated-MYOD1 in order to facilitate polymerase elongation at muscle genes in a manner similar to BRD3-GATA1.

Strikingly, BRD3 and BRD4 were found to exert opposite effects on myogenic differentiation. This observation raises two mechanistic questions. Do BRD3 and BRD4 regulate separate repertoires of target genes with opposing biological functions? Or alternatively, do BRD3 and BRD4 compete for binding at pro-myogenic loci, such that BRD3 antagonizes the function of BRD4? Accordingly, ChIP-seq studies performed in the context of prostate cancer have shown a high degree of overlap in the sites occupied by each BET protein, but also subsets of uniquely bound loci^47^. It will be important to perform similar experiments in the context of muscle differentiation to determine the functional relationship between BRD3 and BRD4. Conversely, histone-peptide array analysis revealed that the recombinant BET bromodomain motifs exhibit differing preferences for patterns of lysine acetylation^48^. The physiological relevance of these findings is not clear, especially given the high degree of structural similarity between the BET bromodomain motifs, and the potential role of non-bromodomain protein regions in determining target site specificity.

Paradoxically, while BRD4 is required for efficient myogenesis, and becomes enriched at the *Myog* promoter during differentiation, its protein expression levels progressively decline in response to differentiation cues. This observation is similar to the differentiation-associated reduction in expression of components of the transcriptional machinery that are essential for the activation of myogenic loci^37^. It is therefore possible that the association of BRD4 with the genome becomes more specialized upon the initiation of differentiation. To achieve this reorganization, BRD3/4 occupancy may hypothetically be modulated by association with further protein cofactors, or by post-translational modifications, which alter BET protein acetyl-lysine binding preferences under differentiation conditions.

Regardless of the mechanistic details, specific inhibition of BRD3 may be of therapeutic benefit in the case of acquired/inherited muscle-wasting disorders (analogous to the promotion of muscle growth in the case of Myostatin pathway blockade)^49^, or alternatively to promote myogenic differentiation at the expense of proliferation in the case of rhabdomyosarcoma. In several respects, BET proteins are attractive drug targets. The bromodomain motif, consisting of a bundle of α-helices, forms a hydrophobic pocket which recognizes acetylated lysine residues but can equally serve as a druggable cavity that accommodates a small molecule inhibitor. BETi binding to the bromodomain motifs therefore disrupts the interaction between the BET protein and acetylated histone and non-histone proteins, thereby directly interfering with BET protein function. As a result, pan-BETi compounds have been demonstrated to be potent anti-tumor agents in the context of NUT midline carcinoma^22^, acute myeloid leukemia, mixed lineage leukemia^24^, MLL-fusion leukemia^50^, multiple myeloma^51^, lung adenocarcinoma^25^, glioblastoma^52^, and neuroblastoma^53^. However, given the wide variety of cellular processes involving BET proteins^14–17, 27–29, 31–35, 54, 55^ (including myogenic differentiation described here) prolonged treatment with conventional pan-BETi compounds may induce unwanted off-target effects in a therapeutic context. Similarly, the use of pan-BETi compounds may mask the subtleties of BET gene function in a research setting. These observations, together with the opposing roles of BRD3 and BRD4 presented here, underline the need for BET-protein specific inhibitors. However, the high degree of structural/sequence similarity between the bromodomain motifs across all family members means that the development of a bromodomain-binding small molecule capable of discriminating between the BET proteins represents a significant technical challenge. Alternative strategies for specific BET family member inhibition include small molecules that bind to non-conserved regions outside of the bromodomain motifs (e.g. a phosphorylation cluster of BRD4)^34^, or oligonucleotide-based gene silencing approaches.

## Methods

### Cell Culture

C2C12 cultures (ATCC, Manassas, VA, USA) were maintained in GM; DMEM High Glucose (GE Healthcare, Wauwatosa, WI, USA), 15% Fetal Bovine Serum (FBS), 1% Penicillin/Streptomycin (P/S). For differentiation, 1 × 10^5^ cells (per 24 well multiwell plate) were seeded and maintained in GM for 48 hours. Cells were switched to DM; DMEM, 5% Horse Serum, 1% P/S, for 3 days (unless otherwise stated).

IMR-90 fibroblasts (Coriell Institute, Camden, NJ, USA) were stably transfected with a murine *Myod1* transgene using the Sleeping Beauty Transposon system. IMR-90 cells were propagated in GM; EMEM (GE Healthcare), 10% FBS. MYOD1 expression was induced by treatment with doxycycline (200 ng/ml) for 24 hours in GM. IMR-90 cells were differentiated in DM, EMEM, 2% Horse Serum, 200 ng/ml doxycycline.

Satellite cells were harvested from the hindlimbs of C57BL/6 wild-type mice. Muscles were minced and digested with Collagenase and Dispase. Satellite cells (CD31^−^/CD45^−^/Ter111^−^/CD34^+^/α7Integrin^+^/Sca1^−^) were collected by Fluorescence Activated Cell Sorting. Cells (1 × 10^4^ per 96 well multiwall plate) were cultured on Laminin (Roche, Pleasanton, CA, USA) coated plates, and maintained in GM (50% DMEM Low Glucose (Life Technologies), 50% F-10 (Life Technologies), 1% P/S, 15% FBS, 2.5 ng/ml basic Fibroblast Growth Factor (bFGF, PeproTech, Rocky Hill, NY, USA)). For differentiation, cultures were switched to DM (DMEM Low Glucose, 2% Horse Serum, 1% P/S) for 48 hours. All animal protocols were carried out in accordance with all relevant guidelines and regulations. Animal experimentation protocols were approved by the Sanford Burnham Prebys Medical Discovery Institute Animal Care and Use Committee (IACUC).

Primary human skeletal muscle cells (Lonza, Allendale, NJ, USA) were maintained in GM (F-10 (Life Technologies), 10% FBS, 1% P/S). Cells (1 × 10^4^ per 96 well multiplate well) were cultured on collagen-coated plates. For differentiation, ~80% confluent cultures were switched to DM (DMEM Low Glucose, 2% HS, 1% P/S) for 72 hours.

### Compounds

The epigenetic probe library was obtained from the Structural Genomics Consortium (University of Oxford, http://www.thesgc.org/chemical-probes/epigenetics, version as available in May 2014). Additional compounds were obtained from commercial sources; (−)-JQ1 (Fisher Scientific, Hampton NH, USA), and SB 203580 (Sigma-Aldrich, St. Louis, MO, USA). All compounds were resuspended in DMSO to a final concentration of 10 mM for long-term storage. For treatments, a ten-fold dilution series of stock solutions were prepared in DMSO (10 mM to 1 µM). Immediately before usage, these stocks were diluted 1:1,000 in cell culture media and mixed by vortexing before being added to cell culture plates. In this way, compounds were added at final concentrations ranging from 1 nM to 10 µM as required, and the final concentration of DMSO (0.1% v/v) was consistent between all treatment groups regardless of compound concentration.

### Immunofluorescence

Cells were washed with Phosphate-Buffered Saline (PBS), fixed with 4% paraformaldehyde (Santa Cruz Biotechnology, Dallas, TX, USA), and permeabilized with 0.25% Triton X-100 (Sigma-Aldrich). Cells were then blocked with 5% Bovine Serum Albumin (BSA, Jackson Immuno Research, West Grove, PA, USA) before sequential incubation with appropriate primary and secondary antibodies (Table S1). Nuclei were stained with Hoechst 33258 (Life Technologies). Myogenic Index was defined as the percentage of nuclei within MHC-positive cells. Fusion Index was defined as the percentage of nuclei within MHC-positive myotubes containing at least 3 nuclei.

Microscopy images were acquired using an Olympus IX71S1F-2 (Olympus, Waltham, MA, USA) inverted fluorescence microscope, with a 10×/0.30 objective lens and a Lumenera Infinity 3 camera (Lumenera Corporation, Ottawa, Canada). Infinity Analyze software v6.5.2 (Lumenera) was used to collect images and subsequent image handling was performed in ImageJ.

### Western Blot

Protein samples were harvested in RIPA buffer (Thermo Scientific, Waltham, MA, USA) supplemented with Complete Protease Inhibitor cocktail and PhosSTOP Phosphatase Inhibition cocktail (both Sigma). Total protein was quantified by Micro BCA Protein Assay (Thermo Scientific) and samples denatured in Laemmli-SDS-Sample buffer (BioWorld, Dublin, OH, USA) supplemented with 10 mM DTT and heated at 95 °C for 10 minutes. Equal amounts of protein were separated by SDS-PAGE and blotted onto nitrocellulose membranes (GE Healthcare, Pittsburgh, PA, USA) by electrotransfer. Membranes were blocked with 5% Milk (BD, Franklin Lakes, NJ, USA) or 5% BSA as required. Blocked membranes were incubated sequentially with primary and secondary antibodies (Supplementary Table S1) and washed with Tris-Buffered Saline with 0.1% Tween 20 (TBST).

### RT-qPCR

All Reverse Transcriptase-quantitative Polymerase Chain Reaction ﻿(RT-qPCR) studies were designed to comply with the MIQE guidelines where applicable or practical^56^. RNA was extracted from cell cultures using RNeasy mini columns (Qiagen, Valencia, CA, USA). 200 ng of total RNA was reverse transcribed using the High Capacity cDNA Synthesis kit (Life Technologies) and cDNA was subsequently diluted 1:10 in nuclease-free water. qPCR was performed on a LightCycler 96 real-time instrument (Roche) using FastStart Essential DNA Green Master Mix (Roche) in 20 µl reactions. The following universal cycling conditions were used; hot start 95 °C for 5 minutes, followed by 40 cycles of 95 °C denaturation for 5 seconds and 60 °C annealing/extension for 30 seconds. Specificity of amplification was confirmed by melt curve analysis. Gene-of-interest expression was normalized to the geometric mean of two stably expressed reference genes (*Rplp0* and *Rpl10*)^57^. PCR reaction efficiencies were determined empirically by amplification curve analysis using LinRegPCR^58^ and expression ratios calculated using the Pfaffl method^59^ as described in detail previously^60^. Details of primers are provided in Supplementary Table S2. RT-qPCR assays were validated by analyzing standard curves of serial cDNA dilutions in order to demonstrate assay linearity, dynamic range, and single amplicon reaction products (Supplementary Fig. S6).

### Cell Viability and Proliferation Assays

Cell viability was determined by MTS assay using the CellTiter 96 AQueous One Solution Cell Proliferation Assay kit (Promega, Fitchburg, WI, USA). Apoptosis/necrosis was assessed by staining with Annexin V-FITC and propidium iodide as described previously^61^. Proliferation was determined using the Click-iT EdU Alexa Fluor 555 Imaging Kit (Life Technologies). Briefly, cells were pulsed for 2 hours with 10 µM EdU to metabolically label newly synthesized DNA. Cells were fixed and permeabilized before the conjugation of Alexa Fluor 555 to the incorporated EdU by azide-alkyne cycloaddition (click chemistry).

### Cell Cycle Analysis by Flow Cytometry

C2C12 cells were cultured for 48 hours in GM and then switched to DM. Experimental groups were treated with DMSO vehicle, (−)-JQ1, (+)-JQ1 (1 µM), or DM only (untreated) as appropriate. After 12 hours, cells were fixed with 80% ethanol and incubated on ice for 30 minutes. Cells were washed twice with PBS and then incubated with staining solution (100 µg/ml propidium iodide (Sigma-Aldrich), 200 µg/ml RNase A (Thermo Scientific), 0.2% Triton X-100 in PBS) for 30 minutes at 37 °C. DNA staining was then assessed by flow cytometry using a FACSCalibur flow cytometer (BD Biosciences, La Jolla, CA, USA) counting 30,000 events within the target (singlet) cell gate. Data were collected using CellQuestPro (BD Biosciences) and the percentage of cells in each phase of the cell cycle was determined using ModFit LT (Verity Software House Topsham, ME, USA).

### RNA Interference

C2C12 myoblasts were seeded in GM as described above. After 24 hours, cultures were treated with siRNAs (100 nM) complexed with Lipofectamine RNAiMAX (Life Technologies) according to manufacturer’s protocols. Transfection complexes were prepared in Opti-MEM (Life Technologies). The siRNAs used were ON-TARGETplus mouse siRNAs SMARTpools against *Brd2* (14312), *Brd3* (67382), *Brd4* (57261) and the Non-targeting control pool (D-001810-10-05) (all GE Dharmacon, Lafayette, CO, USA).

### Chromatin Immunoprecipitation

C2C12 cells were cultured in 15 cm dishes (6 dishes per GM condition or 3 dishes per DM condition). Cells were washed with PBS, crosslinked with 1% formaldehyde for 10 minutes (Fisher Scientific), and the crosslinking terminated by the addition of glycine (to 0.125 M). Cells were then washed with PBS containing protease inhibitors and phosphatase inhibitors; Complete Protease Inhibitor cocktail, PhosSTOP Phosphatase Inhibition cocktail, 0.1 mM Phenylmethanesulfonyl fluoride (all Sigma), and collected by scraping. Cell pellets were frozen at −80 °C until ready for further processing. Pellets were resuspended in Hypotonic Buffer (50 mM Tris-HCl pH 8, 5 mM EDTA, 10% glycerol, and inhibitors as above) and incubated on ice for 5 minutes. Cells were collected by centrifugation, resuspended in RIPA Buffer (50 mM Tris-HCl pH 8, 5 mM EDTA, 150 mM NaCl, 0.5% SDS, 1% NP-40, 0.5% sodium deoxycholate, and inhibitors), and then incubated on ice for 10 minutes. Genomic DNA was subsequently sheared by sonication on ice using a Misonix Sonicator 3000 (Misonix Inc, Farmingdale, NY, USA) equipped with a microtip probe; power setting 3 (6 W), 6 cycles, 15 seconds on, 60 seconds off). Chromatin was sonicated to a size of 200–500 base pairs as confirmed by agarose gel electrophoresis, and stored at −80 °C until ready for further analysis. Sheared chromatin was diluted 1:5 in RIPA Buffer without SDS (with inhibitors) to reduce the sample SDS concentration to 0.1%, and the protein content of each sample determined by Micro BCA Protein Assay (Thermo Scientific). 300 µg of each chromatin sample was incubated overnight in 300 µl total volume with appropriate antibodies (Table S1) at 4 °C with rotation. Normal rabbit Immunoglobulin G (Santa Cruz Biotechnology) was used as negative control antibody. Immune complexes were precipitated by incubation with 1.2 µg Protein A Dynabeads (Life Technologies) per sample (pre-blocked with 20 mg/ml BSA) for 3 hours with rotation at 4 °C. The magnetic beads were then washed as follows; four washes with RIPA containing 0.1% SDS (inhibitors were added to the first wash only), one wash with LiCl Wash Buffer (10 mM Tris-HCl pH 8, 1 mM EDTA, 0.25 M LiCl, 1% NP-40, 1% sodium deoxycholate), and two washes with TE Wash Buffer (10 mM Tris-HCl pH 8, 1 mM EDTA). Immune complexes were eluted by incubation in ChIP Elution Buffer (1% SDS, 0.1 M NaHCO_3_) for 15 minutes at 65 °C. Crosslinking was reversed by incubation with RNase A (50 µg/ml) overnight at 65 °C, followed by incubation with Proteinase K (100 µg/ml) for 1 hour at 45 °C. DNA was recovered using QIAquick PCR Purification columns (Qiagen) and eluted in 50 µl nuclease-free water as according to manufacturer’s instructions. ChIP DNA was further diluted 1:5 in water and 2 µl DNA used per 20 µl qPCR reaction. ChIP signals were reported as the percentage of input samples (non-immunoprecipitated) after background (IgG) subtraction. Primers for ChIP-qPCR are listed in Supplementary Table S2.

### Statistics

Statistical analysis was performed in GraphPad Prism 5 (GraphPad Software Inc, La Jolla, CA, USA). Comparisons between two groups were tested using an unpaired *t*-test. Comparisons between multiple groups were tested using one-way ANOVA and Bonferroni *post hoc* tests. Differences were considered significant at the *P* < 0.05 level.

## Electronic supplementary material

Supplementary Information
