# Supplementary material for: BRD3 and BRD4 BET Bromodomain Proteins Differentially Regulate Skeletal Myogenesis
Source: Sci Rep. 2017 Jul 21;7:6153. doi: 10.1038/s41598-017-06483-7 (PMC5522382; doi:10.1038/s41598-017-06483-7)
Supplement: Supplementary file 1 — Supplementary Information [file 41598_2017_6483_MOESM1_ESM.pdf]

Supplementary Information

Title
BRD3 and BRD4 BET Bromodomain Proteins Differentially Regulate Skeletal Myogenesis

Authors
Thomas C. Roberts[1,2], Usue Etxaniz[1], Alessandra Dall'Agnese[1], Shwu-Yuan Wu[3,4], Cheng-Ming Chiang[3,4,5], Paul E. Brennan[6], Matthew J.A. Wood[2], and Pier Lorenzo Puri[1,7*]

Author Affiliations
[1] Sanford Burnham Prebys Medical Discovery Institute, Development, Aging and Regeneration Program, La Jolla, CA, 92037, USA
[2] Department of Physiology, Anatomy and Genetics, University of Oxford, South Parks Road, Oxford, OX1 3QX, UK
[3] Simmons Comprehensive Cancer Center, University of Texas Southwestern Medical Center, 5323 Harry Hines Boulevard, Dallas, Texas 75390, USA
[4] Department of Biochemistry, University of Texas Southwestern Medical Center, 5323 Harry Hines Boulevard, Dallas, Texas 75390, USA
[5] Department of Pharmacology, University of Texas Southwestern Medical Center, 5323 Harry Hines Boulevard, Dallas, Texas 75390, USA
[6] Structural Genomics Consortium and Target Discovery Institute, Nuffield Department of Clinical Medicine, University of Oxford, Oxford OX3 7DQ, UK
[7] IRCCS Fondazione Santa Lucia, Rome, Italy
*To whom correspondence should be addressed: Prof. Pier Lorenzo Puri, Development, Aging and Regeneration Program, Sanford Burnham Prebys Medical Discovery Institute, La Jolla, CA

Suplementary Tables

Primary Antibody	Host	ID	Manufacturer	Usage
**IF/WB**				
Anti-MYOG	Mouse mAb	F5D	DSHB	1:10
Anti-MHC	Mouse mAb	MF 20	DSHB	1:10
**WB**				
Anti-MYOD1	Rabbit pAb	M-318	Santa Cruz (sc-760)	1:500
Anti-α-Tubulin	Mouse mAb	B-5-1-2	Sigma (T5168)	1:3000
Anti-BRD2	Rabbit mAb	D89B4	CST (5848)	1:1000
Anti-BRD3	Mouse mAb	2088C3a	Santa Cruz (sc-81202)	1:200
Anti-BRD4	Rabbit mAb	EPR5150(2)	AbCam (ab128874)	1:1000
Anti-BRD4-pS484/488	Rabbit pAb		Chiang Lab	1:2000 (BSA)
Anti-BRD4-(N)	Rabbit pAb	CW152	Chiang Lab	1:2000
Anti-CCND1A	Rabbit mAb	EP272Y	EMD Millipore (04221)	1:500
**IF/WB Secondary Abs**				
Anti-Mouse IgG Alexa 488	Goat pAb	A10680	Life Technologies	
Anti-Mouse-HRP	Goat	170-5047	BioRad	1:2000
Anti-Rabbit-HRP	Goat	170-5046	BioRad	1:2000
**ChIP**				
Anti-H3K27Ac	Rabbit pAb	39133	Active Motif	5 µg
Anti-BRD3	Rabbit pAb	61489	Active Motif	5 µg
Anti-BRD4	Rabbit pAb	E2A7X	CST (13440)	10 µg
Normal IgG	Rabbit pAb		Santa Cruz (sc-2027)	10 µg

Table S1

List of antibodies used in this study.

All primary antibodies listed were used for western blot. The monoclonal antibodies anti-MYOG and anti-MHC were also used for immunofluorescence and were obtained from the Developmental Studies Hybridoma Bank, created by the NICHD of the NIH and maintained at The University of Iowa, Department of Biology, Iowa City, IA 52242.

Target	Forward	Reverse
**RT-qPCR**		
Myh1	CCAAAGCCAACAGTGAAGTG	TGGCGTTCACAGCTTCTAC
Myog	CGATCTCCGCTACAGAGG	CGCGAGCAAATGATCTCCT
Ckm	CAAACCCACAGACAAGCATAAG	AGAGTGTAACCCTTGATGCTG
Brd2	GCCCTTCTATAAGCCAGTGG	AAACTCCTGTGCATCCCG
Brd3	AGATGGATAGCCGAGAGTACC	GCAAACCTCATCTCAAACACATC
Brd4	CAAAAGGAAGAGGACGAGGG	TTGATGCTTGAGTTGTGTTTGG
Rplp0	AAGCAAAGGAAGAGTCGGAG	CCAGACCGGAGTTTTAAGAGAAG
Rpl10	TCATGTCCATCCGAACCAAG	GCATTAAACTTGGTGAAGCCC
Ccnd1a	GCCCTCCGTATCTTACTTCAAG	GCGGTCCAGGTAGTTCATG
Cdkn1a	CTTGCACTCTGGTGTCTGAG	GCACTTCAGGGTTTTCTCTTG
**ChIP**		
Myog promoter	GCTCAGGTTTCTGTGGCGTT	CCAACTGCTGGGTGCCAT
Sox2 TSS	GATTGGCCGCCGGAAAC	CTCTTCTCTGCCTTGACAACTC

Table S2

List of qPCR assays used in this study.

All sequences are 5′ to 3′.

Supplementary Figures

a

Bromodomain inhibitors	
(+)-JQ1	BET
PFI-1	BET
GSK2801	BAZ2A/B
SGC-CBP30	CREBBP/EP300
I-CBP112	CREBBP/EP300
PFI-3	SMARCA2/4,PBRM1
Bromosporine	Pan-Bromodomain
C646	EP300

Histone methyltransferase inhibitors	
SGC0946	DOT1L
GSK343	EZH2
UNC1999	EZH2
UNC0638	G9a/GLP
UNC0642	G9a/GLP
A-366	G9a/GLP
PFI-2	SETD7
LLY-507	SMYD2

Lysine demethylase inhibitors	
GSK-J1/J4*	JMJD3,UTX,JARID1B
GSK854	LSD1
IOX1	2-OxoGlutarate Oxygenases
IOX2	PHD

Histone deacetylase inhibitors	
CI-994	HDACs
LAQ824	HDACs

Other	
Olarparib	PARP

b

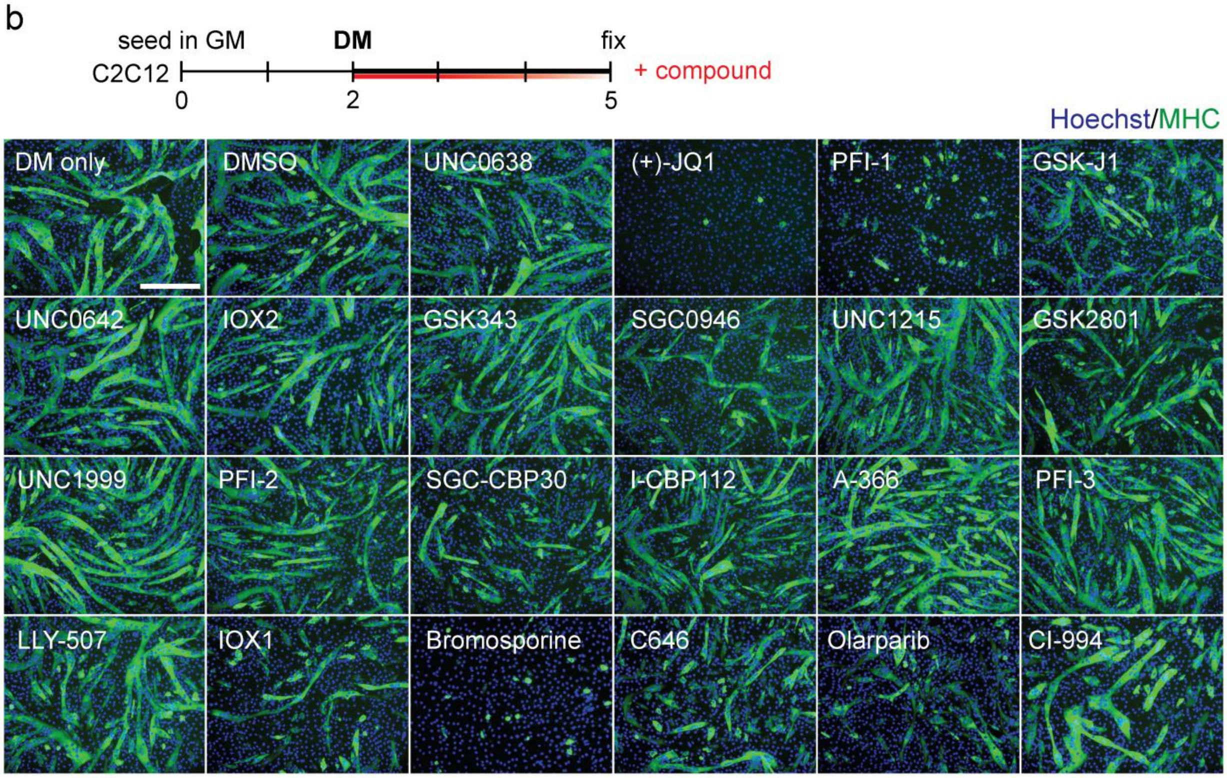

Figure S1

Screen of small molecule epigenetic probes in C2C12 myoblasts.

(a) List of small molecule epigenetic probes used in this study and their target proteins. (b) C2C12 cells were cultured in Growth Media (GM) for two days and then switched to Differentiation Media (DM) for a further three days. Small molecule probes or DMSO vehicle control were added at the same time as the switch to DM. Myogenic differentiation was determined by immunofluorescence staining for Myosin Heavy Chain (MHC) and nuclei stained with Hoechst. The following compounds were used at a final concentration of 10 µM; (+)-JQ1, GSK-J1 (Note: GSK-J4 is the prodrug form of this compound), IOX2, SGC0946, UNC1215, GSK2801, UNC1999, PFI-2, PFI-3, IOX1, C646, Olarparib. The remaining compounds were used at a final concentration of 1 µM; UNC0638, PFI-1, UNC0642, GSK343, SGC-CBP30, I-CBP112, A-366, LLY-507, Bromosporine, CI-994. The choice of concentration was based on preliminary measurements of acute cytotoxicity. LAQ824 was found to be highly toxic at all concentrations tested. Images were taken at 10× magnification, scale bars indicate 50 µm.

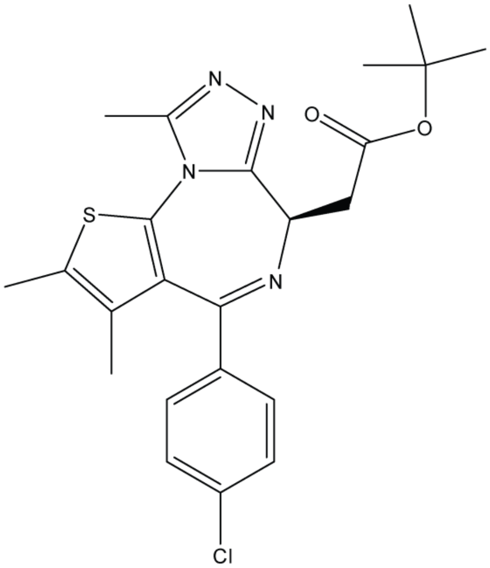

(-)-JQ1

Inactive stereoisomer

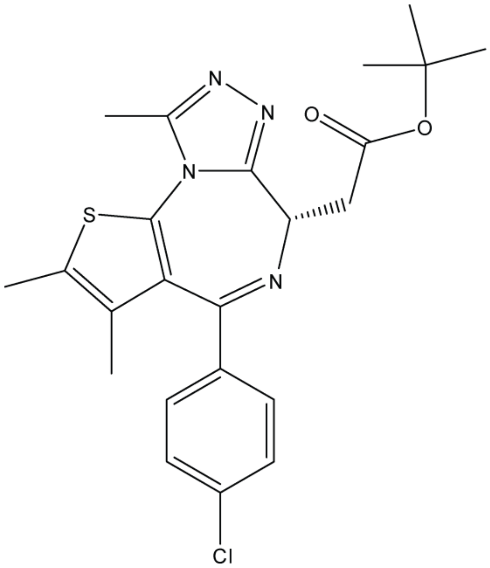

(+)-JQ1

BRD2
BRD3
BRD4
BRDT

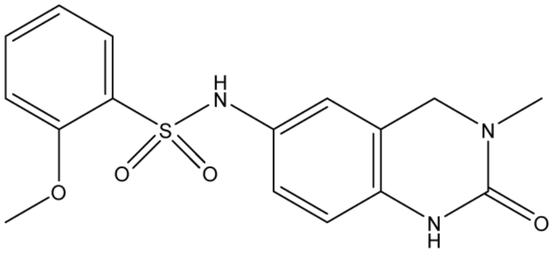

PFI-1

BRD2
BRD3
BRD4
BRDT

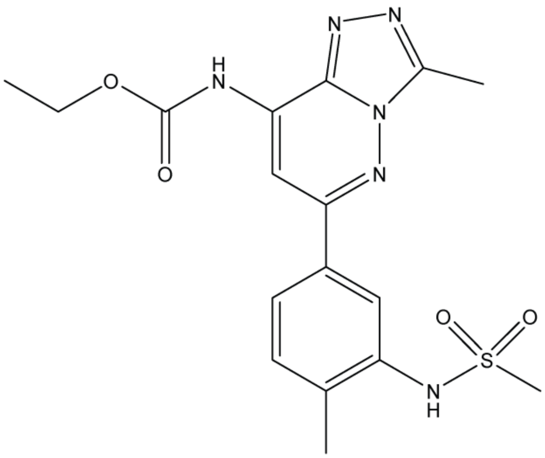

Bromosporine

BRD2
BRD3
BRD4
BRDT
CECR2
TAF1
BRD9
CREBBP

Figure S2

Chemical structures of BETi compounds used in this study.

(-)-JQ1 and (+)-JQ1 are triazolothienodiazapine stereoisomers that differ only in the orientation of the butyl ester group at the chiral C6 position of the diazepine ring. (+)-JQ1 is specific for the BET bromodomain proteins (BRD2, BRD3, BRD4 and BRDT) whereas (-)-JQ1 is the biologically inactive enantiomer. PFI-1 is a dihydroquinazoline which is also specific for BET bromodomain proteins. Bromosporine is a triazolopyridazine, and a pan-Bromodomain inhibitor that targets the BET family in addition to several other bromodomain-containing proteins.

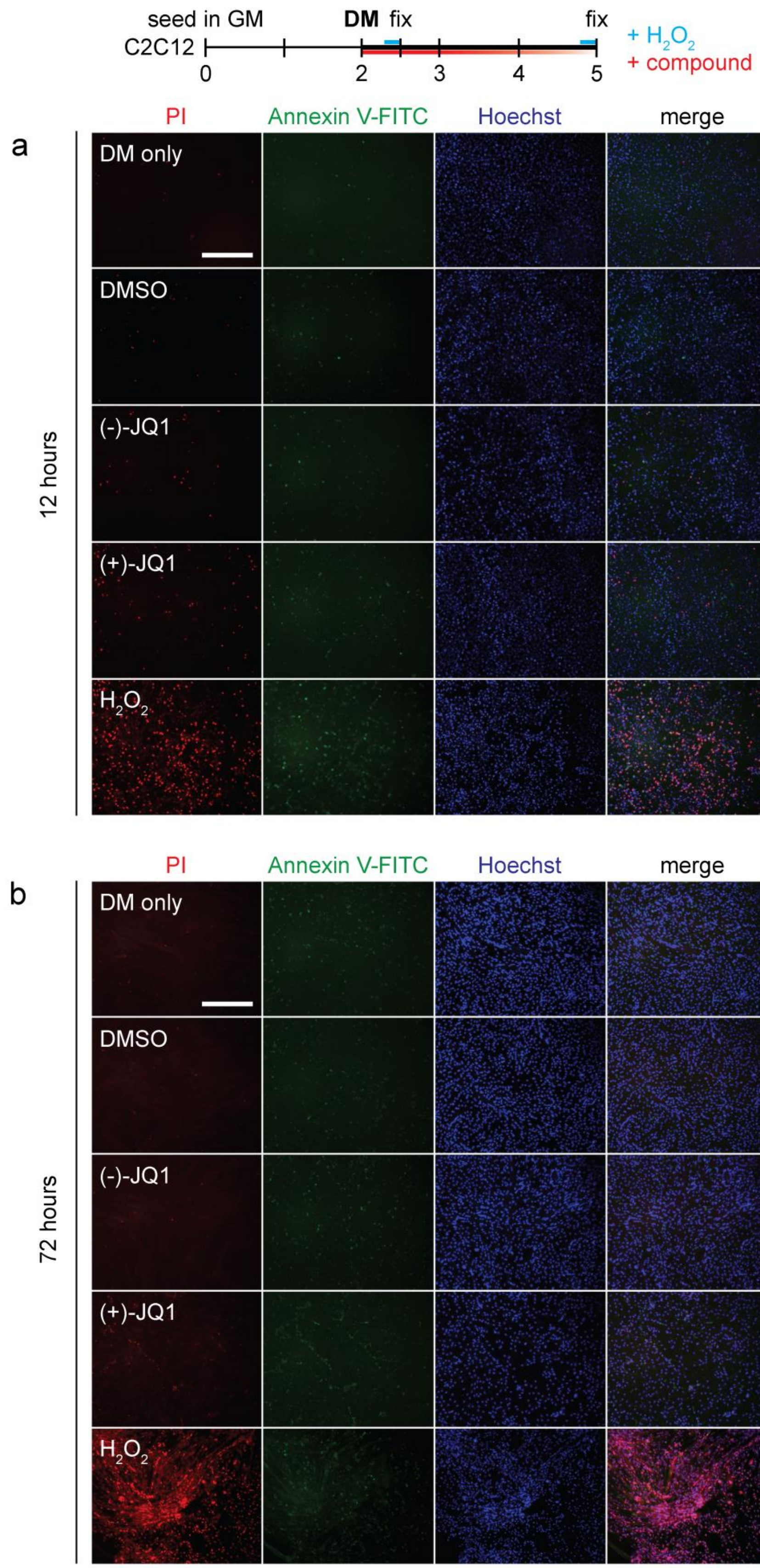
seed in GM
DM
fix
fix
C2C12
0
2
3
4
5
+ H_2O_2
+ compound
PI
Annexin V-FITC
Hoechst
merge
a
12 hours
DM only
DMSO
(-)-JQ1
(+)-JQ1
H_2O_2
b
72 hours
DM only
DMSO
(-)-JQ1
(+)-JQ1
H_2O_2

Figure S3

(+)-JQ1 induces minimal apoptosis in C2C12 cultures.

C2C12 myoblasts were cultured in Growth Media (GM) and then switched to Differentiation Media (DM) for 3 days. Cultures were treated with 1 µM (+)-JQ1 and compared with cultures treated with DM only (untreated), DMSO vehicle, or (-)-JQ1 negative control enantiomer. Cultures were treated with 8 mM hydrogen peroxide (H_2O_2) for 4 hours prior to fixing as a positive control for apoptosis induction. Cultures were fixed at (a) 12 hours, and (b) 72 hours following compound treatment. All microscopy images were taken at 10× magnification, scale bars indicate 50 µm.

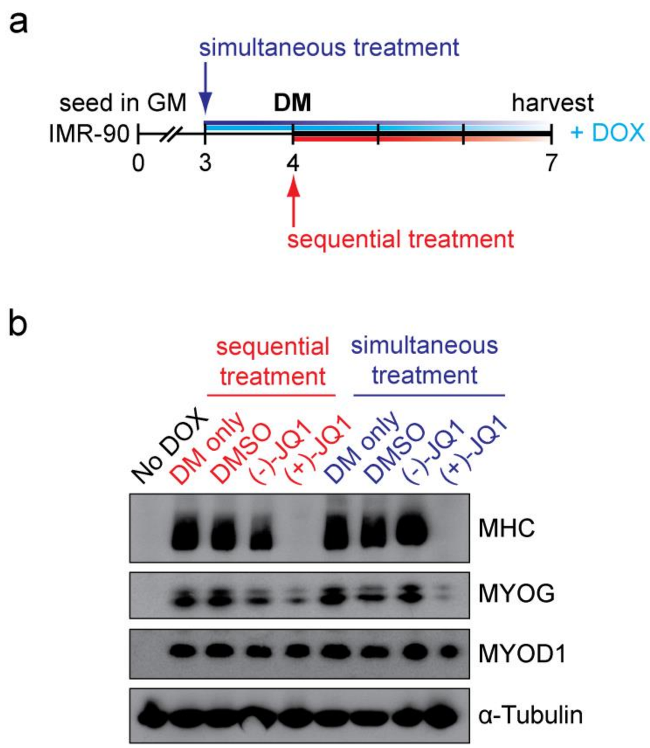

Figure S4

Timing of (+)-JQ1 treatment differential effects MYOG expression.

(a) MYOD1 expression was induced in IMR-90 fibroblasts with doxycycline (DOX) and (+)-JQ1 for 24 hours before switching to DM. Cultures were treated with (+)-JQ1 or controls concurrent with DOX induction (simultaneous treatment) or concurrent with the switch to DM (sequential treatment). (b) After 3 days in DM, MHC, MYOG and MYOD1 expression were assessed by Western blot.

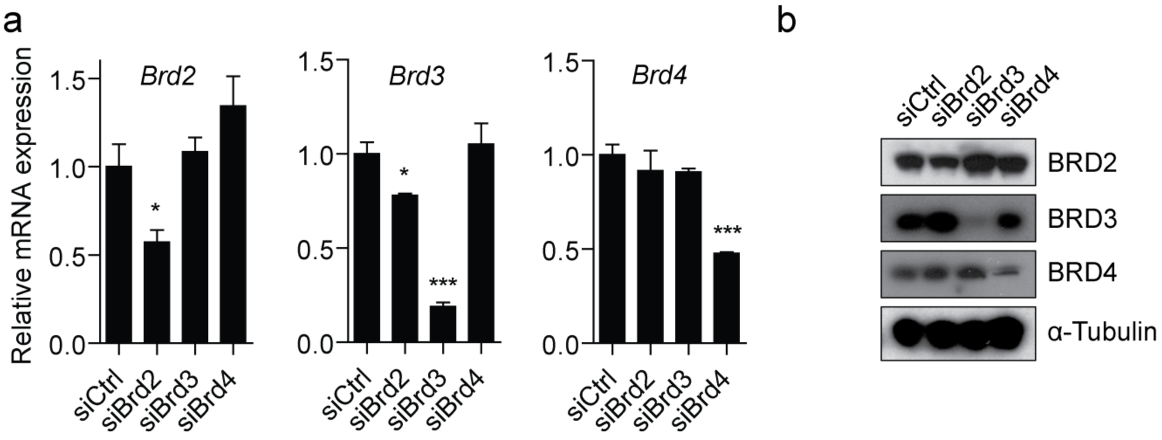

Figure S5

RNAi knockdown efficiency in differentiating C2C12 cultures.

Validation of siRNA-mediated gene knockdown in C2C12 cultures treated with 100 nM siBrd2, siBrd3 or siBrd4. siCtrl is a non-specific control siRNA by (a) RT-qPCR, and (b) Western blot. All values are mean+SD, n=3, *P<0.05, ***P<0.001. Statistical significance was determined by one-way ANOVA with Bonferroni *post hoc* test, and comparisons to the siCtrl control group reported.

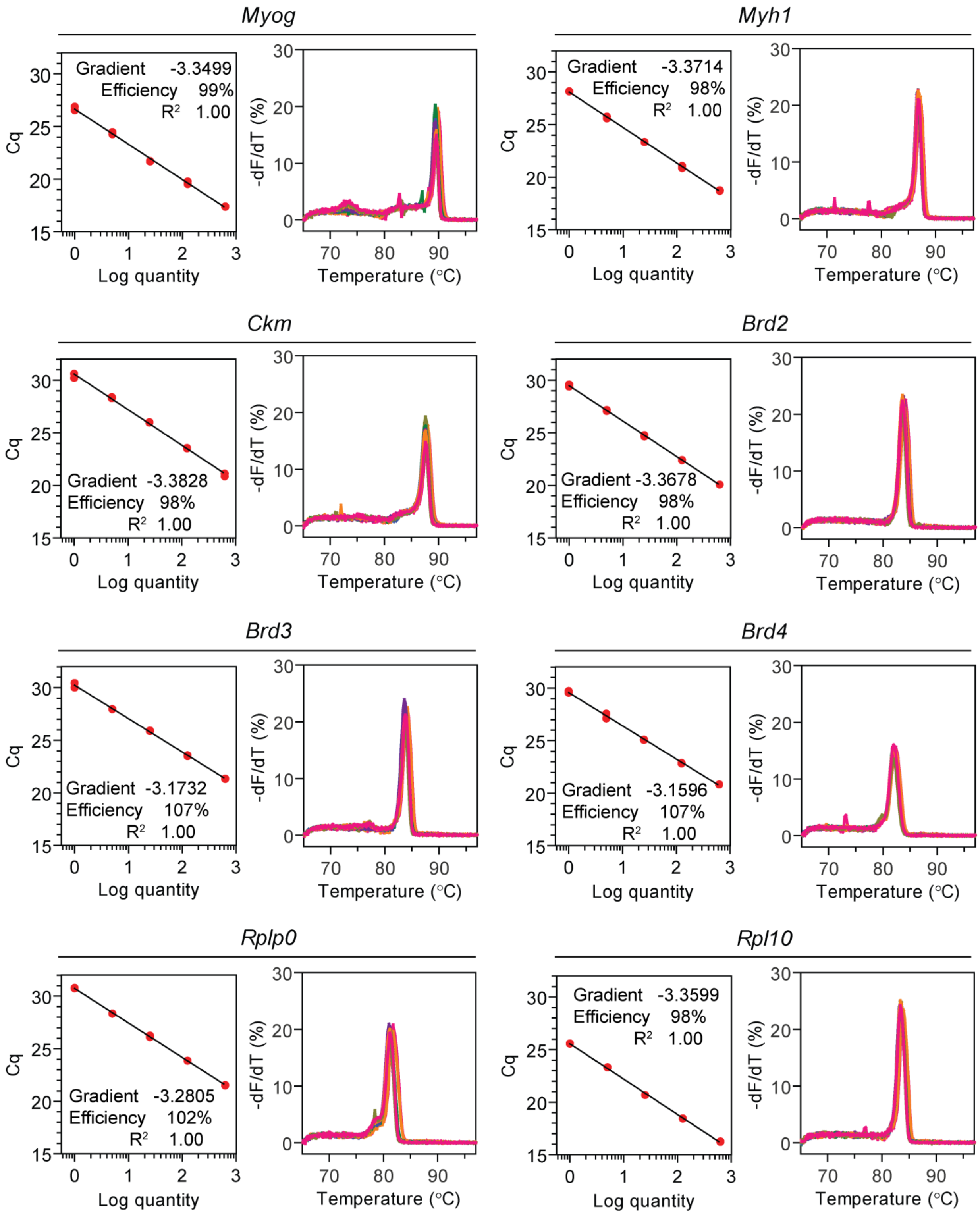
Myog
Gradient -3.3499
Efficiency 99%
R² 1.00
Cq
Log quantity
-dF/dT (%)
Temperature (°C)
Myh1
Gradient -3.3714
Efficiency 98%
R² 1.00
Ckm
Gradient -3.3828
Efficiency 98%
R² 1.00
Brd2
Gradient -3.3678
Efficiency 98%
R² 1.00
Brd3
Gradient -3.1732
Efficiency 107%
R² 1.00
Brd4
Gradient -3.1596
Efficiency 107%
R² 1.00
Rplp0
Gradient -3.2805
Efficiency 102%
R² 1.00
Rpl10
Gradient -3.3599
Efficiency 98%
R² 1.00

Figure S6

Validation of RT-qPCR assays used in this study.

Standard curves of serially diluted cDNA were generated to demonstrate assay linearity, dynamic range, and specificity of amplification for RT-qPCR assays used in this study.
